# The beneficial effects of the tobacco hydroperoxide lyase pathway in whitefly host adaptation

**DOI:** 10.1007/s44297-023-00019-4

**Published:** 2023-12-12

**Authors:** Wenhao Deng, Ping Li, Chao Liu, Songshen Hu, Yanzhen Tian, Yinquan Liu

**Affiliations:** 1 Ministry of Agriculture Key Lab of Molecular Biology of Crop Pathogens and Insects, Institute of Insect Sciences, Zhejiang University, Hangzhou, 310058 China; 2 State Key Laboratory for Managing Biotic and Chemical Threats to the Quality and Safety of Agro-Products, Key Laboratory of Biotechnology in Plant Protection of Ministry of Agriculture and Zhejiang Province, Institute of Plant Virology, Ningbo University, Ningbo, 315211 China; 3 Key Laboratory of Horticultural Plant Growth, Development and Quality Improvement, Ministry of Agriculture, Department of Horticulture, Zhejiang University, Hangzhou, 310058 China

**Keywords:** HPL pathway, Whitefly, GLV, Plant defense

## Abstract

Plants respond to herbivore attack by emitting complex mixtures of volatile compounds to repel herbivores or attract predators and parasitoids. In this study, however, we revealed that tobacco green leaf volatiles (GLVs) were beneficial compounds for whitefly adaptation to host plants. Our study indicates that GLVs are closely associated with plant susceptibility to whiteflies. Whitefly infestation elevated the transcript of the *hydroperoxide lyase* (HPL) gene, one gene responsible for catalyzing the synthesis of C6 or C9 aldehydes from fatty acid hydroperoxides. Overexpression of *HPL* increased the emission of GLVs (hexanal, 1- hexanal, trans-2- hexanal and cis-3- hexanal) and resulted in improved performance for whiteflies. Exogenous application of GLVs promoted whitefly survival and fecundity and increased the plant's attraction to whiteflies. Thus, our study provides new insights into the role of the HPL pathway and GLVs in influencing herbivore adaptation to host plants.

**Supplementary Information:**

The online version contains supplementary material available at 10.1007/s44297-023-00019-4.

## Introduction

In nature, plants defend themselves against diverse biotic and abiotic stimuli by a myriad of sophisticated induced defenses [6, 47]. These defense responses depend on intricate signaling cascades that include peptide signaling, phytohormones and others, in which oxylipin signals play crucial roles [39]. Oxylipin signals are derived from two main pathways: the allene oxide synthase (AOS) pathway, including 12-oxo-phytodienoic acid (OPDA), jasmonic acid (JA) and methyl jasmonate (MeJA), collectively known as jasmonates (JAs), and the hydroperoxide lyase (HPL) pathway, which generates C6 aldehydes and their corresponding derivatives, collectively named green leaf volatiles (GLVs) [2]. The role of GLVs in plant defense is more intricate in comparison to the JAs pathway. GLVs are composed of several different aldehydes, alcohols, and esters, with the most common compounds being cis-3-hexenal, trans-2-hexenal, hexanal, and trans-3-hexenol. They act as vital messengers in communicating between neighboring plants and initiating direct and indirect defense [30, 35, 43].

Under normal conditions, GLVs are produced at relatively low levels by undamaged plants but are often formed and released in response to wounding, pathogen infection or herbivore attack [2, 31, 45]. Although their emission is instantaneous, it can be sustained by repetitive wounding, which often occurs during damage and is also controlled by the plant circadian clock [20, 24, 37]. GLVs have armed plants with various functions. Firstly, they are known to have a direct inhibitory effect on pathogens [3, 14, 28, 33], although recently, it has been found that *Arabidopsis thaliana* with enhanced E-2-hexenal emission was more susceptible to the pathogen *Pseudomonas syringae* pv. Tomato DC3000 [32]. Secondly, GLVs appear to function as insect repellants or attractants by influencing the performance of several herbivore species [19, 31] and could also be used as feeding stimuli for some lepidopteran larvae, such as *Manduca sexta* and *M. quinquemaculata* and the generalist insect *Spodoptera exigua* [13]. Third, GLVs play an important role in the recruitment of foraging predators or parasitoids to the plant [1, 4, 7, 22]**.** Fourth, GLVs can induce the expression of defense-related genes and metabolic changes [10, 17, 40]. Taken together, GLVs are a class of organic compounds that act as molecular clues in plant defense against biotic stress and damage.

The emission of GLVs from plants attacked by pests is a typical response. GLVs might exert positive or negative effects on herbivores. Potato plants expressing *hydroperoxide lyase* (HPL) in an antisense orientation produced relatively lower amounts of GLVs, leading to enhanced aphid performance [38]. The phloem-feeding herbivore brown planthopper (BPH) preferred to feed on *hpl3-1* plants over WT plants, and female adults laid more eggs on *hpl3-1* plants [36]. However, the mass of individual striped stem borer (SSB) larvae was reduced in *hpl3-1* mutant plants. GLV-deficient *N. attenuata* plants slowed down the development of the chewing herbivore *Manduca sexta* larvae and decreased attractiveness to three lepidopteran herbivores, the closely related specialist species *Manduca sexta* and *M. quinquemaculata* and the generalist *Spodoptera exigua* [13]. Field experiments with tobacco (*N. attenuata*) showed that GLV-producing wild-type plants were more attractive to flea beetles (*Epitrix hirtipennis*) compared to plants with reduced *HPL* expression (as-*hpl*) [13]. The diverse effects of GLVs on different herbivores highlight the complexity of plant–herbivore interactions and the importance of understanding the specific context in which GLVs function in plant defense.

The whitefly *Bemisia tabaci* (Hemiptera: Aleyrodidae), a typical phloem-feeding insect, is a species complex including invasive cryptic species such as the Middle East-Asia Minor (MEAM1) [8], which causes great economic losses around the world not only by direct sucking of plant sap but also by indirect transmission of devasting plant viruses, such as African Cassava Mosaic virus [34]. Here, we found that tobacco GLVs are vital for host adaptation of whiteflies. Through integration of over-expression and virus-induced gene silencing (VIGS) approaches, we observed that higher accumulation of GLVs in tobacco plants overexpressing the *HPL* gene (*Hydroperoxide lyase*) was favorable for whitefly infestation, as indicated by higher survival rates and increased egg laying on *HPL-OE* plants. Lower accumulation of GLVs in plants with *hpl* and *adh* silencing resulted in reduced whitefly performance. Additionally, our findings demonstrated that the emission of GLVs can affect the preference of whiteflies for the host. Overall, we provide solid evidence for the significant contribution of GLVs in shaping plant–insect interactions.

## Materials and methods

### Plants

The tabacco *Nicotiana tabacum* cv. NC89 was used in this study. To reduce gene expression, 500 bp sequences from *HPL* or *ADH* were amplified and inserted into the 2mDNA1 vector. The plasmids were transformed into *Agrobacterium tumefaciens* strain EHA105. The detailed construction method is shown below. Tobacco plants with 2–3 leaves were inoculated with HPL-2mDNA1 or ADH-2mDNA1 and cultivated in a greenhouse (light: 06:00–20:00, 14:10 L/D, controlled temperature 25 ± 3 °C, and relative humidity 60–80%) for 3 weeks. Then, the gene transcript abundance was determined, and plants with higher silencing efficiency were selected to perform bioassays. The plants with the empty vector 2mDNA1 were used as controls. For construction of *HPL* gene-overexpressing plants, a pCHF3 recombinant plasmid containing the full-length HPL gene was transformed into plants. Calluses with kanamycin resistance were cultivated into seedlings and verified by PCR and qRT‒PCR.

### Whitefly culture

The whitefly species *Bemisia tabaci* MEAM1 (*mtCOI* GenBank accession no. GQ332577) from Zhejiang, China, was collected from eggplants in Rui’an (27°48′20′′N, 120°39′57′′E) in September 2008. In the experiments, whiteflies were maintained on healthy tobacco plants in insect-proof cages (40 cm × 50 cm × 50 cm) in climate-controlled rooms at 25 ± 1 °C, a 14 L:10D light cycle, and 60–80% RH.

### Subcellular localization analysis

Full-length HPL was inserted into a modified pCambia1305-RFP vector and transformed into *Agrobacterium tumefaciens* strain GV3101*.* The images were taken 3 days after inoculation. The CLSM data were collected at the Bioimaging Center, State Key Laboratory for Managing Biotic and Chemical Threats to the Quality and Safety of Agro-products, Institute of Plant Virology, Ningbo University, Ningbo, China.

### Analysis of HPL activity

HPL activity was analyzed with a plant HPL ELISA kit. HPL activity was determined using a standard curve equation (Y = 0.02534*X—0.01446, R^2^ = 0.9984), followed by normalization with respect to a control sample.

### Analyses of HPL-derived metabolites

Volatiles and endogenous HPL-derived metabolites were extracted and analyzed by GC–MS as described previously [5]. Leaf samples were collected and frozen in liquid nitrogen. The materials were ground, and a weighed amount of the sample was introduced into a 4 mL screw-top Supelco vial containing 500 μL of 1% NaCl. The vial was then rapidly capped with the screw top having a polytetrafluoroethylene/silicone septum and incubated for 30 min in a water bath at 50 °C. A 60 μm polydimethylsiloxane (PDMS)-coated solid phase micro extraction (SPME; Supelco) was used to measure the aldehydes released from the plant tissue. Measurements were performed in triplicate. The headspace was sampled for 30 min with PDMS-SPME and analyzed by GC‒MS. GC‒MS analysis was performed using a Hewlett and Packard 6890 series gas chromatograph coupled to an Agilent Technologies 5973 network mass selective detector. An HP-5MS column (30 m × 0.25 mm, 0.25 μm film thickness) was used with He (37 kPa) as the carrier gas. The GC oven temperature was programmed as follows: 5 min 40 °C, ramp to 225 °C at 15 °C/min and no hold time. Mass spectra in electron impact mode were generated at 70 eV. Injection was performed by thermal desorption of the SPME in the injector at 200 °C using the splitless injection mode. The compounds were identified by comparing the GC retention times and mass spectra with those of authentic reference compounds. The headspace was analyzed as described above, and peak areas (mass-to-charge ratios 82 and 98) were determined. The aldehydes were quantified subsequent to careful preparation of calibration curves with different standards.

### Host plant suitability assays

Five female and five male whitefly adults emerging within three days were collected from uninfected tobacco plants and released into a clip cage that was secured to the abaxial surface of a plant leaf (third to fifth leaf from the top). Each plant was equipped with three clip cages, and the experiment was replicated thirty times. The number of adult whiteflies and eggs laid by whiteflies on each plant were counted after 7 days. Based on these data, the survival rate of adult whiteflies as well as the number of eggs laid per female per day were determined to assess host plant suitability.

### GLVs treatments

Plants were grown for 22 days under the conditions mentioned above before being treated with GLVs. For the GLVs treatment, 10 plants in single pots were placed into airtight glass insect cages and treated with 500 nmol of (z)-3-hexenol in lanolin. Control plants were smeared with the same volume of pure lanolin paste. The host plant suitability assays were conducted as described above.

### Choice experiments

The choice experiments were performed as described previously [21]. Two plants with similar sizes and the same leaf numbers were pretreated with or without GLVs and placed in a cage (30 × 30 × 30 cm). Two hundred adult whiteflies were captured, placed on ice for 1 min, and then released onto the petri dish in the middle of the two plants. Fifteen minutes after insect release, the settled whiteflies were recaptured, and the number on each of the two plants was recorded.

### RNA extraction and RT‒PCR analysis

Total RNA was extracted from leaves with TRIzol (Invitrogen, USA). cDNA was synthesized from 1 μg of total RNA using the SYBR® PrimeScript RT‒PCR Kit II (Takara Biotechnology, Dalian, China). qRT‒PCR was performed using a Bio-Rad CFX96™ Real-Time System (Bio-Rad, CA, USA). Eight independent biological samples and three technical repetitions were used. The average threshold cycle (Ct) was calculated per sample. The relative expression levels were calculated with the 2^−ΔΔCT^ method. The *glyceraldehyde-3-phosphate dehydrogenase* (*GAPDH*) gene served as an internal control [46]. The gene-specific primers used for examining transcript abundance are listed in Table 1.

**Table 1:** Primers used in this study

Gene	GenBank accession	Primer sequences (5’→3’)	Application
*HPL*	DQ129870	AATGGCGAAAATGATGAGC GAATTGTACGGACGGGAAG	qRT‒PCR
*ADH* *GAPDH*	XM_016637202 Z72488	GCTTCTAGGGTCATTGGCAT CATCTCAGCAATGACCTGCT GCAGTGAACGACCCATTTATCTC AACCTTCTTGGCACCACCCT	qRT‒PCR qRT‒PCR
*HPL* *ADH*	DQ129870 XM_016637202	TGCTCTAGAACAAATAGCACCCCAAT CGCGGATCCTTGACAAATCTTGGCCTTTG CGCGGATCCGTGCCATACTGATGTTTACT TGCTCTAGACACAACTTAGAACGCAAA	*HPL* VIGS *ADH* VIGS
*HPL*	DQ129870	CGCGGATCCATGTCCACAATAATGGCG TGCTCTAGATCAACTGGCTTTTTTCACAG	HPL-OE
*HPL*	DQ129870	AGCTCTAGAATGTCCACAATAATGGCG CATGGATCCACTGGCTTTTTTCACAGA	HPL-RFP

### Virus-induced gene silencing (VIGS) and gene overexpressing plant assays

Gene-specific primers for VIGS of the *HPL* (Genebank accession no. DQ129870) and *ADH* (Genebank accession no. XM_016637202) genes were designed, which contain *BamH*I or *Xba*I enzyme digestion sites in the forward and reverse primers, respectively (Table 1). Fragments of *HPL* and *ADH* were amplified from tobacco cDNA. The PCR product was cloned and inserted into the *Xba*I-*BamH*I-digested pBIN2mDNA1 plasmid, yielding a gene-silencing vector as previously described [18]. After sequencing confirmation of the fidelity for the insertion, the gene-silencing vector was transformed into *A. tumefaciens* strain EHA105 by electroporation. With the medical syringe, approximately 0.2 mL of *A. tumefaciens* cultures (OD600 = 0.8–1.0) that carry TbCSV as a helper virus and 2mDNA1-*HPL*/*ADH* constructs were co-infiltrated into the stem of each plant at the three-to-four true-leaf stage (VIGS silenced plants). Empty-vector plants were used as the control and were inoculated with *A. tumefaciens* cultures carrying TbCSV and pBIN2mDNA1. For gene overexpression, the *HPL* gene coding region was inserted into the pCHF3 vector and transformed into *A. tumefaciens* strain EHA105 by electroporation. After that, leaf disc transformation of tobacco was performed, and the callus was selected by kanamycin. The F_0_ plants were used to determine the gene expression levels and host plant suitability assays. As described above, all plants were cultivated in a greenhouse under the same conditions. Total RNA was isolated from the third leaf from the top, and then the silencing efficiency was evaluated by qRT‒PCR.

### Data analysis

All percentage data (adult survival) were arcsine square root transformed before statistical analysis. Statistical significance was evaluated using one-way ANOVA Student's *t*-test at a 0.05 level followed by least significant difference tests for comparisons of survival, the number of eggs laid and gene expression. All data analyses were conducted using SPSS 20.0. Statistics (IBM, Armonk, NY, USA).

## Results

### HPL contributes to plant adaptation in whiteflies

**Fig. 1:**
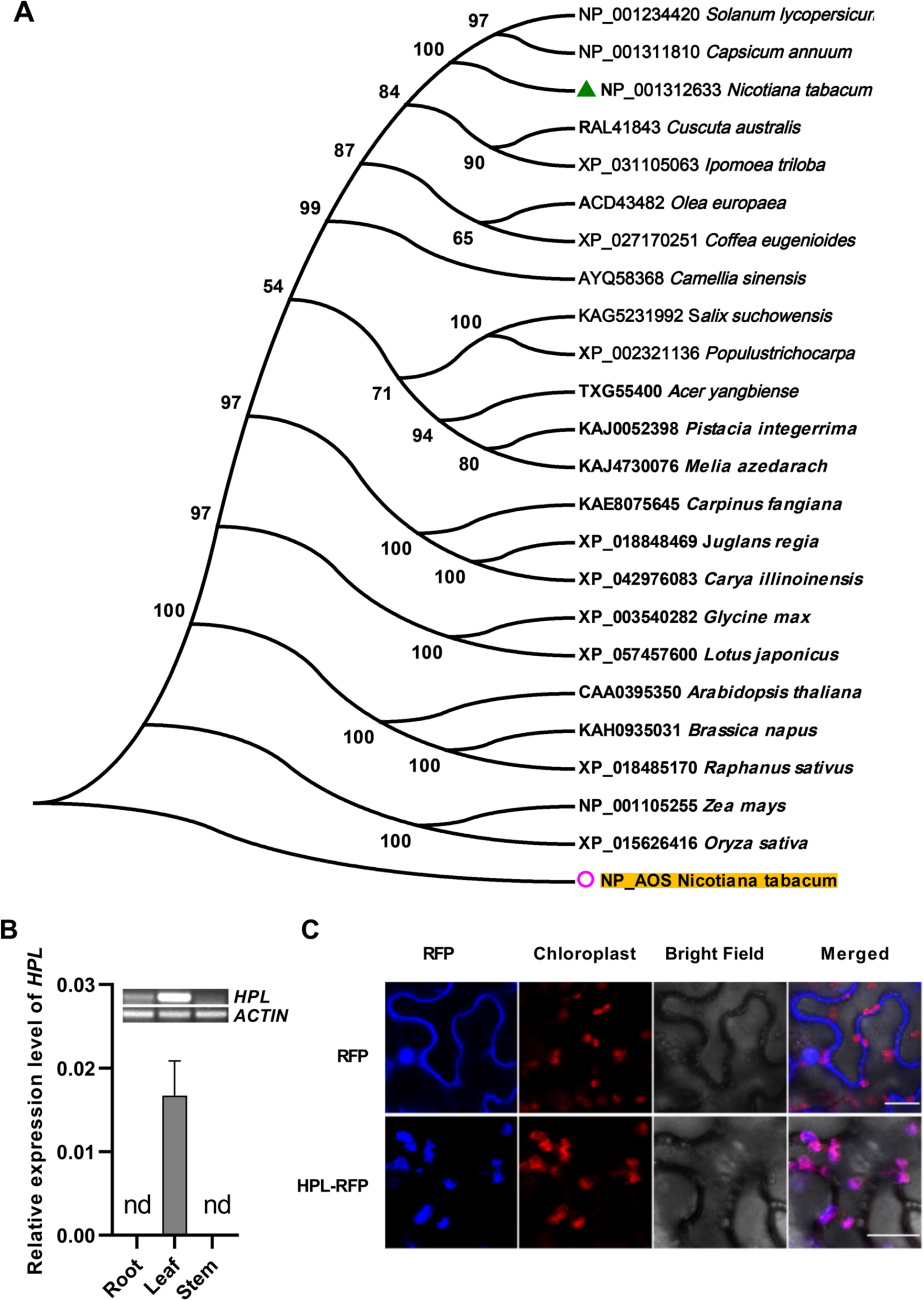
Characterization of HPL in tobacco. **A** Phylogenetic analysis of HPL among different plant species. The neighbor-joining method was used, and a bootstrap analysis of 1000 replicates was used. The resulting bootstrap values are shown at the nodes in the cladogram. The phylogenetic analysis was performed with MEGA5 software. Serial numbers of the proteins used in the figure for different plant species are marked before the respective plant names. AOS was identified as the most homologous protein and served as the outgroup for the evolutionary tree. **B** Transcripts of the *HPL* gene in roots, stems and leaves determined by qRT‒PCR and RT‒PCR. nd indicates that no Ct values were determined by qRT‒PCR. The PCR cycle for RT‒PCR was 45. **C** Subcellular localization of the HPL protein. HPL was fused with RFP (red fluorescent protein) to observe the subcellular localization, while the chloroplast was marked with its autofluorescence

Hydroperoxide lyase (HPL), one of the important enzymes for GLVs synthesis, cleaves the C–C bond adjacent to the hydroperoxy group in the products of the LOX pathway, resulting in the formation of C6 or C9 aldehydes that can undergo isomerization or dehydrogenation [27]. The RNA-seq data obtained from our laboratory [25] showed that the transcript level of the *HPL* gene was significantly increased following whitefly infestation (Data were not shown). This observation suggests that *HPL* might be responsive to whitefly resistance. Evolutionary analysis of the *HPL* gene in tobacco indicated that it was present as a single copy in the genome (Fig. 1A). To gain a deeper understanding of the expression patterns of the *HPL*, we conducted a quantitative real-time PCR (qRT‒PCR) experiment and revealed that its expression was notably higher in leaves than in roots and stems (Fig. 1B). Furthermore, our investigation into the subcellular localization of the HPL protein demonstrated that it was primarily expressed in the chloroplasts of plant cells (Fig. 1C).

**Fig. 2:**
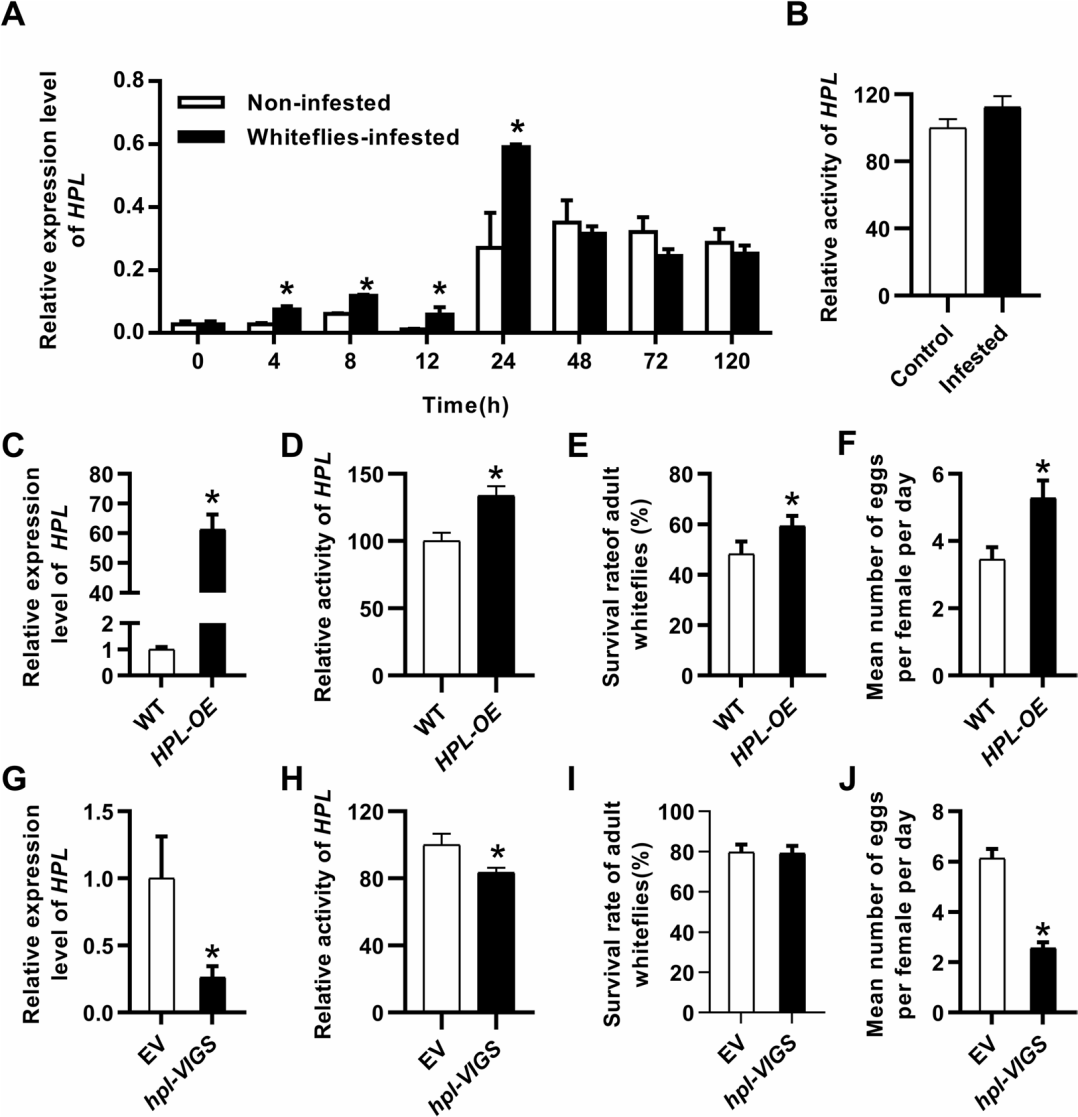
HPL is associated with plant defense against whiteflies. **A** Relative transcript level of *HPL* to GADPH (glyceraldehyde-3-phosphate dehydrogenase) in whitefly infested and control plants at the indicated time points. **B** Enzyme activity of HPL 24 h after whitefly infestation. The HPL activity for control plants was set as 100%. **C**, **D** Relative transcript level and activity of HPL in transgenic plants and the control. Gene expression or enzyme activity in WT was set as 1 or 100%. **E**–**F** Performance of whiteflies on *HPL-OE* plants. The whiteflies were allowed to feed on control and transgenic tobacco plants for seven days. The survival rates of the adults (**E**) or the number of eggs/female/day (**F**) on transgenic and control plants were calculated. **G**-**H** Expression and enzyme activity of HPL in *hpl-VIGS* plants. Gene expression or enzyme activity in WT was set as 1 or 100%. **I**-**J** Performance of whiteflies on *hpl-VIGS* plants. Seven days after whitefly feeding, survival rates of whiteflies (**I**) and mean number of eggs laid by per female adult per day (**J**) on *hpl-VIGS* and empty vector-inoculated control plants. Data are shown as the mean ± SE, n = 3 (**B**, **D**, **H**), 8 (**A**, **C**, **G**) or 30 (**E**–**F**, **I**-**J**). Asterisks above the bars indicate significant differences between treatments (*P* < 0.05, Student’s t-test)

To investigate the potential role of HPL in plant defense against whiteflies, we conducted a series of experiments. First, we compared the relative transcript levels of the *HPL* gene in plants with or without whitefly infestation. Remarkably, we observed a significant upregulation of *HPL* gene expression at the early stage of whitefly infestation (Fig. 2A), suggesting that HPL might play a crucial role in plant resistance to whiteflies. We also observed a slight increase in HPL activity after whitefly infestation (Fig. 2B). Then, we generated transgenic tobacco plants overexpressing the *HPL* gene (*HPL-OE*). No obvious phenotypic differences in growth were observed between the *HPL-OE* plants and wild-type plants. The transcript level of *HPL* in these transgenic plants was markedly higher, approximately 60 times more than that in wild-type plants, which led to a remarkable increase in enzyme activity (Fig. 2C, D). The survival rate of whiteflies fed *HPL-OE* plants was significantly higher than that of whiteflies fed control plants (Fig. 2E). Furthermore, female adults presented increased fecundity, laying approximately 1.53-fold more eggs per day compared to those feeding on wild-type plants (Fig. 2F). To further validate these findings, a bioassay on *HPL* gene silencing (*hpl-VIGS*) and control plants was performed. Seven days after releasing adult whiteflies to the plants, the survival rate of whiteflies on *hpl-VIGS* plants was slightly lower than that on empty-vector-inoculated plants, although the difference was not statistically significant (Fig. 2G-I). However, the mean number of eggs laid per day per female on *hpl-VIGS* plants was significantly lower (Fig. 2J).

Taken together, these results suggest that HPL plays a critical role in determining the suitability of host plants to whiteflies.

### HPL-mediated GLVs biosynthesis is associated with plant defense

**Fig. 3:**
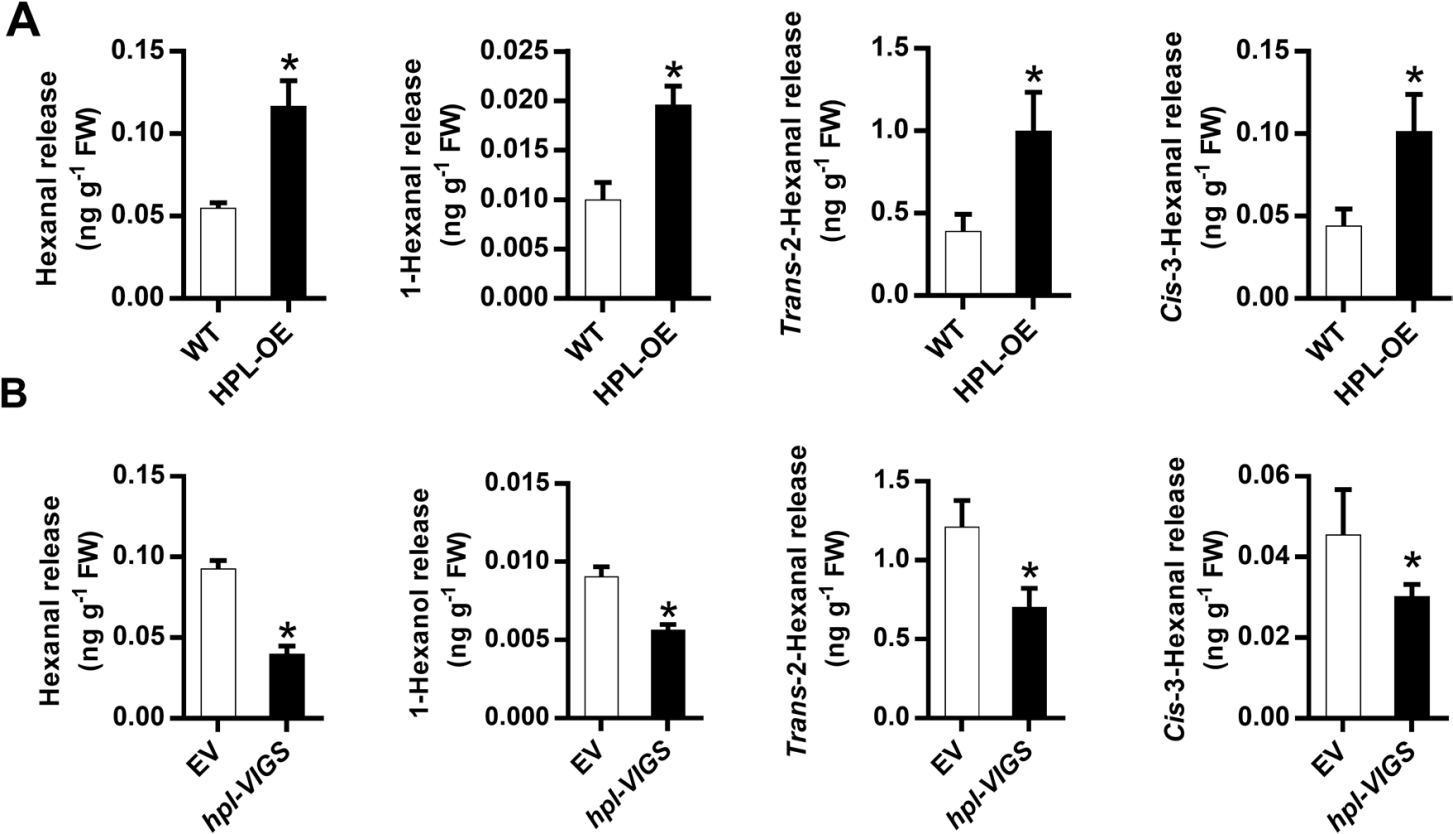
HPL mediates GLVs synthesis. Amounts of Hexanal, 1-Hexanal, Trans-2- Hexanal and Cis-3- Hexanal (cis-3-hexen-1-ol) in control, *HPL*-overexpressing (*HPL-OE*) or *hpl-VIGS* (**B**) plants were measured. The data shown are the mean ± SE. Asterisks above the bars indicate significant differences between treatments (*P* < 0.05, Student’s t test)

As the *HPL* gene encodes a GLV biosynthetic enzyme, manipulating its expression through overexpression or silencing led to alterations in its catalytic activity. To validate the impact of HPL overexpression or silencing on GLVs release, we quantified the amounts of GLVs with high accumulation after insect attack, including hexanal, 1-hexanal, trans-2-hexanal, and cis-3-hexanal, in the plants. The results revealed that in the HPL-overexpressing (*HPL-OE*) plants, the content of all the indicated GLVs compounds was markedly increased, reaching approximately two times that of the control plants (Fig. 3A). In contrast, silencing of the *HPL* gene led to a significant reduction in the release of GLVs (Fig. 3B). All the data support the conclusion that HPL plays a critical role in manipulating the GLV aldehyde pools in the plant.

**Fig. 4:**
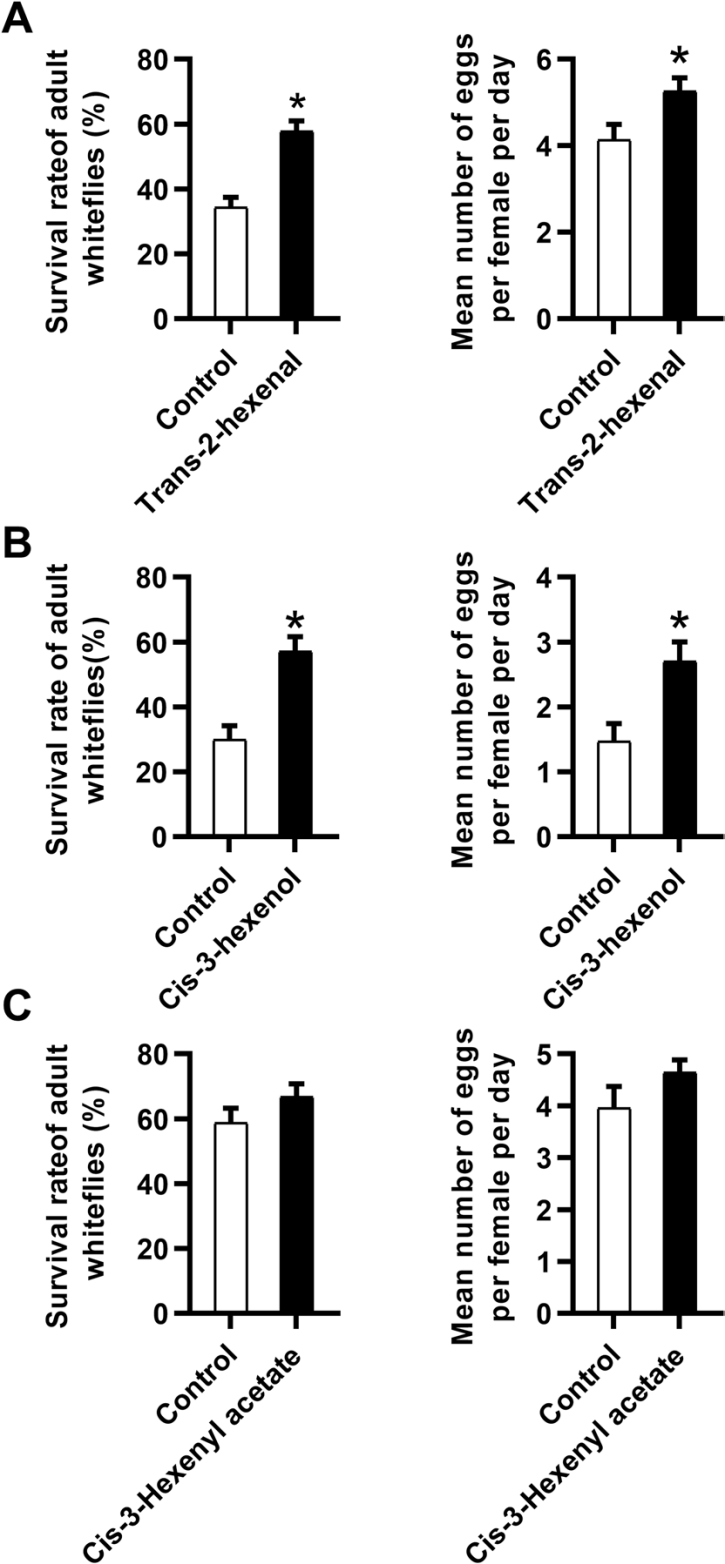
Exogenous application of GLVs could facilitate whitefly performance. Seven days after whitefly feeding, the survival rate of the adults and the number of eggs laid by per female per day on tobacco plants that were individually treated with 500 nmol of trans-2-hexenal (**A**), cis-3-hexenol (**B**) or cis-3-haxenyl acetate (**C**) were analyzed. The data shown are the mean ± SE, *n* = 30. Asterisks above the bars indicate significant differences between treatments (*P* < 0.05, Student’s t-test)

To directly investigate the role of GLVs on whitefly performance, we conducted an exogenous application experiment with three representative GLVs. Both 500 nmol/L trans-2-hexenal and cis-3-hexenol treatment led to reduced plant defense against whiteflies. Whiteflies exhibited higher survival rates and laid more eggs on plants treated with these GLVs (Fig. 4A, B). Although the application of cis-3-hexenyl acetate slightly promoted whitefly performance, the difference was not significant (Fig. 4C). Taken together, our results demonstrated that GLVs negatively regulate plant defense against whiteflies.

### GLVs establish host selection for whiteflies

**Fig. 5:**
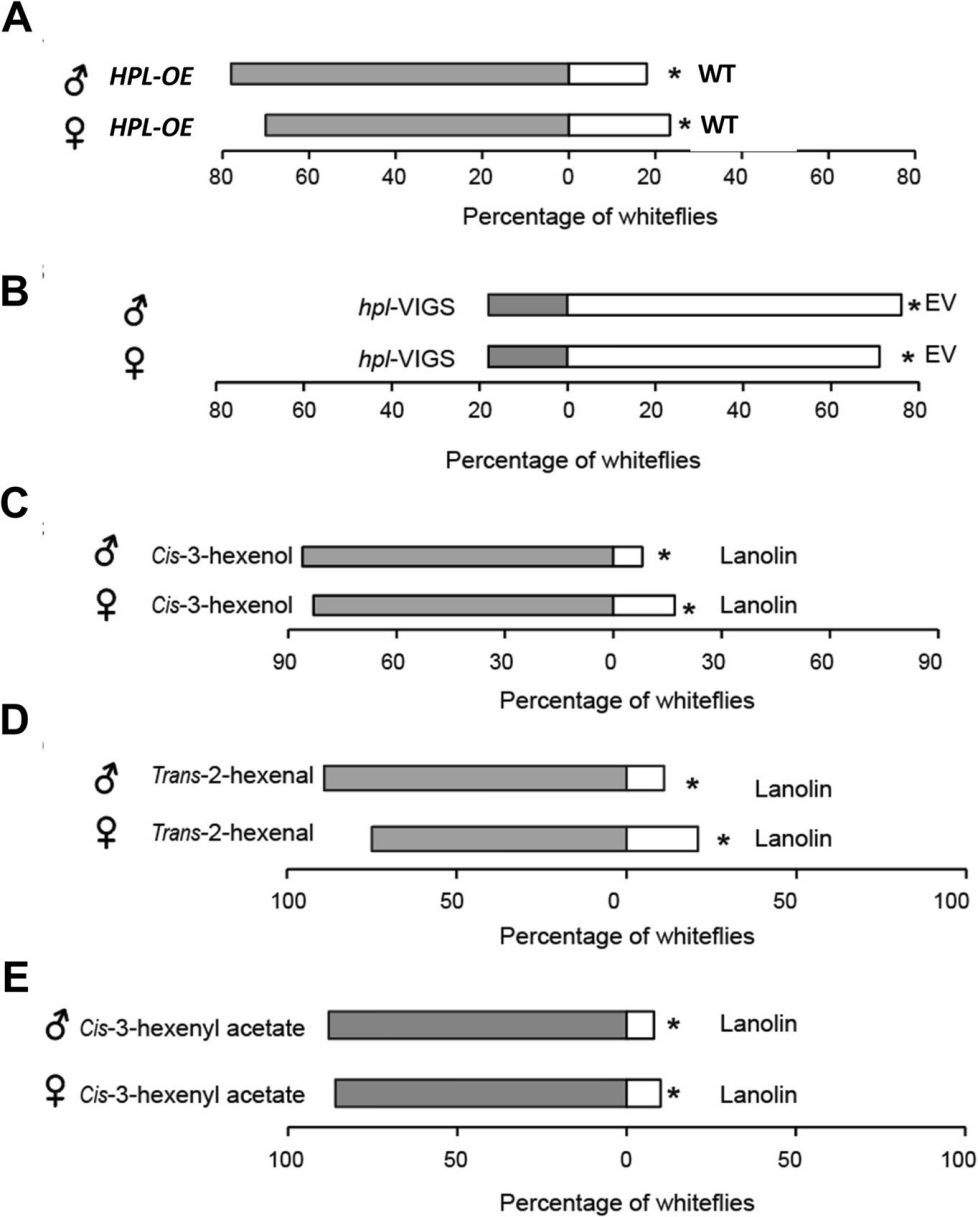
GLVs play an important role in host plant options. **A**, **B** Host choice experiment for adult whiteflies to control, *HPL-OE* or *hpl-VIGS* plants. **C**-**E** Host plant choice experiment for adult whiteflies to control or plants with 500 nmol *cis*-3-hexenol (**C**), *trans*-2-hexenal (**D**) and *Cis*-3-hexenyl acetate (**E**). Whiteflies with no option were also taken into account. All experiments were repeated twice with similar results. Asterisks indicate significant differences (*P* < 0.05, Wilcoxon matched pairs test)

Previous studies have suggested that GLVs could impact the preference of insects for specific plants. Thus, we speculated that changes in GLVsG release might also alter the flying orientations of whiteflies. To test this hypothesis, we examined the choice behavior of female and male whiteflies to wild-type and *HPL-OE* plants and found that whiteflies preferred plants with high GLV accumulation (Fig. 5A). In contrast, *HPL* gene-silenced plants became noticeably less attractive to female and male whiteflies (Fig. 5B). Then, we conducted a comparison of whitefly preferences to cis-3-hexenol-treated or untreated plants. Remarkably, the cis-3-hexenol-treated plants exhibited a significantly higher preference for whiteflies. The percentage of whiteflies on cis-3-hexenol-treated plants was approximately four times higher than that on the control plants (Fig. 5C). Similarly, trans-2-hexenal treatment also had a notable impact on whitefly preference. The choice probability for whiteflies to trans-2-hexenal-treated plants was remarkably high, ranging from 75 to 90% (Fig. 5D). Taken together, these results indicate that plant GLVs are associated with defense against whiteflies.

### Alcohol dehydrogenase contributes to whitefly adaptation to tobacco

**Fig. 6:**
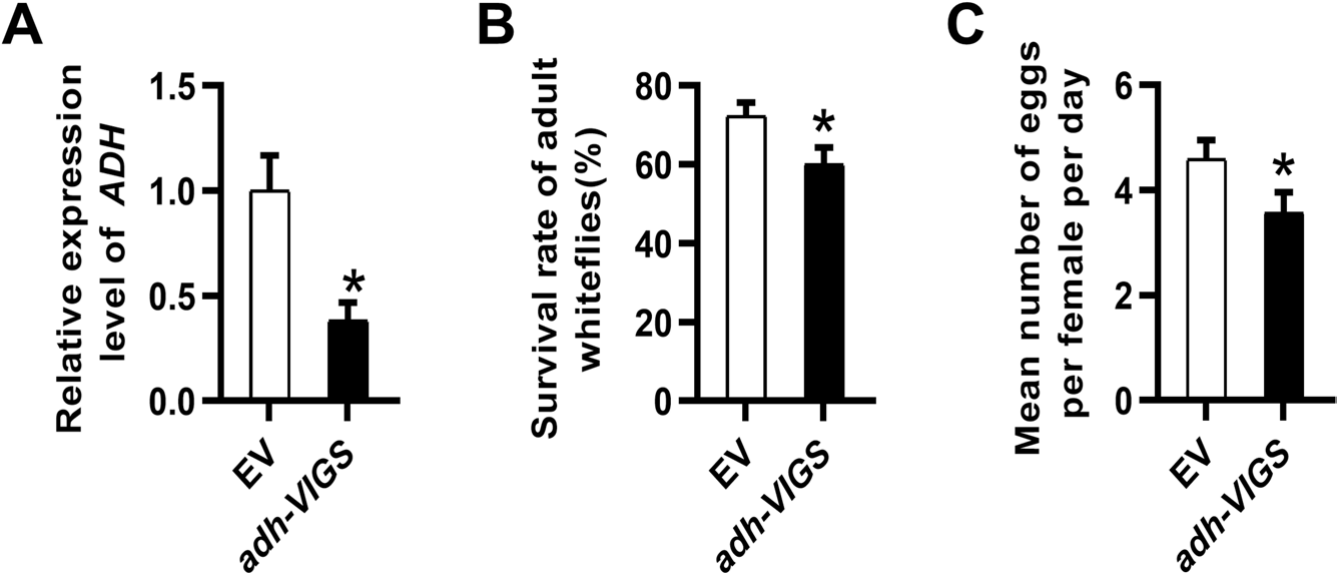
The performance of whiteflies on *adh-VIGS* and empty vector-inoculated plants. **A** Transcripts of the *ADH* gene in *adh-VIGS* plants. Values are means ± SEs (*n* = 8). Seven days after whitefly feeding, the survival rates of the adults (**B**) and the number of eggs by per female per day (**C**) on *adh-VIGS* tobacco plants and empty vector-inoculated plants were compared. The data shown are the mean ± SE, *n* = 30. Asterisks above the bars indicate significant differences between treatments (*P* < 0.05, Student’s t-test)

Metabolites produced by HPL are quite unstable and can be isomerized to the corresponding higher stable alcohol by alcohol dehydrogenase (ADH) [31]. To further confirm the role of the HPL pathway in plant suitability to whiteflies, we investigated the performance of whiteflies on *adh-VIGS* tobacco. Through qRT‒PCR analysis, we observed a significant reduction in the transcript levels of *ADH* in *adh-VIGS* plants, reaching only 38% of the levels found in the empty-vector plants (Fig. 6A). Seven days after whitefly infestation, the survival rate of whiteflies on *adh-VIGS* plants was lower than that on control plants (Fig. 6B). Adult female whiteflies laid a reduced number of eggs on *adh-VIGS* tobacco (Fig. 6C).

## Discussion

As plants are sessile organisms and continuously subjected to a wide array of biotic and abiotic challenges, they have numerous constitutive and inducible defense mechanisms against attackers [6, 16]. Currently, phytohormones, especially JA, salicylic acid and ethylene, have garnered extensive attention [9, 11, 26, 44]. Green leaf volatiles (GLVs) and jasmonates (JAs) are the most widely distributed oxylipin compounds found throughout the plant kingdom. JA has undergone extensive research due to its pivotal role in plant resistance against insect herbivores. Conversely, the role of GLVs in plant defense remains a subject of debate, as they appear to serve varying functions in response to different herbivores. In the present study, we clarified the significant role of the GLV/HPL pathway in plant defense.

GLVs are emitted in trace amounts when plant tissues are intact but are rapidly released within seconds or minutes upon mechanical wounding, herbivore attack, or abiotic stress [13, 37]. Given that GLV/HPL products could be induced by whiteflies and other herbivores [36],Wang et al., 2015), it seems that plants possess the ability to perceive insect infestation and subsequently activate the HPL pathway as part of their defense response. Here, we demonstrated that the HPL pathway plays a negative role in the tobacco response to the sucking insects whiteflies. Whiteflies performed better on *HPL-OE* tobacco plants than on wild-type plants (Fig. 2D-F). Conversely, silencing of *NtHPL* decreased plant adaptability to whiteflies, leading to a reduced survival rate and fecundity of whiteflies (Fig. 2H-J). However, previous reports have shown that the function of HPL in plant direct defense against herbivores can vary widely, possibly depending on the insect or host plant species. For example, *Manduca quinquemaculata*, *Spodoptera exigua* larvae and *Manduca sexta* neonates preferred to choose excised leaves of wild-type *Nicotiana attenuata* (WT), consumed a wider leaf area and grew significantly faster than those on *as-hpl* plants [13]. In contrast, OsHPL3 positively modulates resistance to rice brown planthopper but negatively modulates resistance to the rice striped stem borer and white-backed planthopper [23, 36]. Depletion of *HPL* in potatoes promoted aphid performance [38]. Nevertheless, a study on phloem-feeding insects (aphids: *Myzus persicae*) and insect herbivores (leafminers: *Liriomyza trifolii*) showed no significant differences in performance between WT and *HPL-OE* Arabidopsis plants [5].

As HPL is a key enzyme controlling the synthesis of GLVs, the alteration of its transcript might change the release of GLVs, such as hexanal, 1- hexanal, trans-2- hexanal and cis-3- hexanal. Here, we showed that overexpression of *HPL* promoted the release of GLVs, while silencing of *HPL* reduced their accumulation (Fig. 3). Exogenous application of (*Z*)-3-hexenol also indicated that GLVs play a role in facilitating whitefly performance (Fig. 4). These findings indicate that the individual metabolic components in GLVs could mediate plant resistance to herbivores. Previous studies have also explored the effects of GLVs on insect performance. For example, treatment of tea plants with (*Z*)-3-hexenol reduced the performance of the tea geometrid *Ectropis obliqua* by interfering with the JA and ethylene (ET) pathways [41]. Exposure to (Z)-3-hexenol vapors in glass jars decreased weight gain and oviposition of *B. tabaci*, shortened the total feeding period and phloem ingestion and increased the frequency of stylet puncture in tomato [42].

The plant HPL pathway plays a crucial role in regulating the foraging behavior of insects. Y-tube and greenhouse experiments showed that plastic dummies baited with either single compounds or GLV mixtures were more attractive to tea aphids than hexane-baited controls [15]. The adult emerald ash borer, *Agrilus planipennis*, was more attractive to (*Z*)-3-hexenol-containing purple prism traps [12]. In addition, the mixture of C6-volatiles ((*Z*)-3-hexenol and (*Z*)-3-hexenyl acetate) and benzaldehyde in a natural ratio was found to be more attractive to the female fruit moth *Cydia molesta* [29]. Whiteflies, insects with flying capabilities, can choose host plants based on their preferences. In this study, we found that tobacco plants treated with GLVs became more attractive to whiteflies, further confirming that the HPL pathway plays a vital role in whitefly and plant interactions.

GLV production may itself be used as a signal by plants to coordinate their defensive response to herbivores. Here, we shed light on the complex interactions between plants and insects, particularly the role of GLVs in influencing insect behavior and host plant selection. These insights provide valuable knowledge that may aid in the development of novel strategies for enhancing plant resistance to herbivores.

## Supplementary Information

**Additional file 1: Table 1.** Primers used in this study.

## Data Availability

The data that support the findings of this study are openly available.
