# Supplementary material for: The beneficial effects of the tobacco hydroperoxide lyase pathway in whitefly host adaptation
Source: Crop Health. 2023 Dec 12;1(1):19. doi: 10.1007/s44297-023-00019-4 (PMC12825935; doi:10.1007/s44297-023-00019-4)
Supplement: Supplementary file 1 — Additional file 1: Table 1. Primers used in this study. [file 44297_2023_19_MOESM1_ESM.docx]

**The beneficial effects of tobacco hydroperoxide lyase pathway in whitefly host adaption**

**Wenhao Deng^1#^, Ping Li^1,2#*^, Chao Liu^1^, Songshen Hu^3^, Yanzhen Tian^2^, Yinquan Liu^1*^**

*^1^ Ministry of Agriculture Key Lab of Molecular Biology of Crop Pathogens and Insects, Institute of Insect Sciences, Zhejiang University, Hangzhou 310058, China*

*^2^ State Key Laboratory for Managing Biotic and Chemical Threats to the Quality and Safety of Agro-products, Key Laboratory of Biotechnology in Plant Protection of Ministry of Agriculture and Zhejiang Province, Institute of Plant Virology, Ningbo University, Ningbo 315211, China*

*^3^ Key Laboratory of Horticultural Plant Growth, Development and Quality Improvement, Ministry of Agriculture, Department of Horticulture, Zhejiang University, Hangzhou 310058, China*

# These authors contributed equally

**^*^Correspondence to:** Yinquan Liu & Ping Li; Mailing address: Ministry of Agriculture Key Lab of Molecular Biology of Crop Pathogens and Insects, Institute of Insect Sciences, Zhejiang University, Hangzhou 310058, China; Telephone: 86-571-88982435; Fax: 86-571-88982355; E-mails: [yqliu@zju.edu.cn](mailto:yqliu@zju.edu.cn), [liping6434@126.com](mailto:liping6434@126.com)

**Abstract**

Plants respond to herbivore attack by emitting complex mixtures of volatile compounds to repel herbivores or attract predators and [parasitoids](https://www.sciencedirect.com/topics/agricultural-and-biological-sciences/parasitoids). In this study, however, we reveal that tobacco green leaf volatiles (GLVs) were used as beneficial compounds for whitefly adaption to host plants. Our study indicate that GLVs are closely associated with plant susceptibility to whiteflies. Whitefly infestation elevated the transcript of *hydroperoxide lyase* (HPL) gene, one gene responsible for catalyzing the synthesis of C6 or C9 aldehydes from fatty acid hydroperoxides. Overexpression of *HPL* increase the emission of GLVs (hexanal, 1- hexanal, trans-2- hexanal and cis-3- hexanal) and resulted in the improved performance for whiteflies. Exogenous application of GLVs promoted whitefly survival and fecundity and increased the plant's attraction to whiteflies. Thus, our study provides new insights into the role of HPL pathway and GLVs in influencing herbivore adaption to host plant.

**Introduction**

In nature, plants defend themselves against diverse biotic and abiotic stimuli by a myriad of sophisticated induced defenses ([Chen and Mao, 2020](#_ENREF_6); [Zogli et al., 2020](#_ENREF_41)). These defenses responses depends on a intricate signaling cascades which include peptide signaling, phytohormones and others, in which the oxylipin signals play crucial roles ([Wasternack and Feussner, 2018](#_ENREF_35)). Oxylipin signals derived from two main pathways: the allene oxide synthase (AOS) pathway, including 12-oxo-phytodienoic acid (OPDA), jasmonic acid (JA) and methyl jasmonate (MeJA) collectively known as jasmonates (JAs), and the hydroperoxide lyase (HPL) pathway which generates C6 aldehydes and their corresponding derivatives, collectively named green leaf volatiles (GLVs) ([Ameye et al., 2018](#_ENREF_2)). Role of GLVs in plant defense is more intricate in comparison to JAs pathway. GLVs are composed of several different aldehydes, alcohols, and esters, with the most common compounds being cis-3-hexenal, trans-2-hexenal, hexanal, and trans-3-hexenol. They act as vital messengers in communicating between neighboring plants and initiating direct and indirect defense ([Paudel Timilsena et al., 2020](#_ENREF_26); [Takabayashi and Shiojiri, 2019](#_ENREF_31); [Ye et al., 2019](#_ENREF_37))

Under normal conditions, GLVs are produced at relatively low level by undamaged plants, but are often formed and released in response to wounding, pathogen infection or herbivore attack ([Ameye et al., 2018](#_ENREF_2); [Scala et al., 2013a](#_ENREF_27); [Zhang et al., 2019](#_ENREF_39)). Although their emission is instantaneous, it can be sustained by repetitive wounding, which often occurs during damage, and is also controlled by plant circadian clock ([Joo et al., 2019](#_ENREF_18); [Loughrin et al., 1994](#_ENREF_22); [Turlings et al., 1995](#_ENREF_33)). GLVs have armed plant with various functions. Firstly, they are known to have a direct inhibiting effect on pathogens ([Ameye et al., 2015](#_ENREF_3); [Hammerbacher et al., 2019](#_ENREF_12); [Najdabbasi et al., 2021](#_ENREF_25); [Shiojiri et al., 2006](#_ENREF_29)), although recently it has been found that *Arabidopsis thaliana* with enhanced E-2-hexenal emission were more susceptible to the pathogen *Pseudomona syringae* pv. Tomato DC3000 ([Scala et al., 2013b](#_ENREF_28)). Secondly, GLVs appear to function as insect repellants or attractants by influencing the performance of several herbivore species ([Jones et al., 2022](#_ENREF_17); [Scala et al., 2013a](#_ENREF_27)) and could also be used as feeding stimuli for some lepidopteran larvae, such as *Manduca sexta* and *M. quinquemaculata* and the generalist insect, *Spodoptera exigua* (Halitschke *et al.,* 2004). Third, GLVs play an important role in the recruitment of foraging predators or parasitoids to the plant ([Allmann and Baldwin, 2010](#_ENREF_1); [Brodmann et al., 2008](#_ENREF_4); [Clavijo McCormick et al., 2012](#_ENREF_7); [Liu et al., 2022](#_ENREF_20))**.** Fourth, GLVs can induce the expression of defense-related genes and metabolic changes ([Farag and Pare, 2002](#_ENREF_9); [Hirao et al., 2012](#_ENREF_15); [Xin et al., 2019](#_ENREF_36)). Taken together, GLVs are a class of organic compounds acting as the molecular clues in plant defense against biotic stress and damage.

Emission of GLVs of plants attacked by pests is one of the typical responses. GLVs might exert positive or negative effects on herbivores. Potato plants expressing *hydroperoxide lyase* (HPL) in an antisense orientation produced relatively lower amounts of GLVs, leading to enhanced aphid performance ([Vancanneyt et al., 2001](#_ENREF_34)). The phloem-feeding herbivore brown planthopper (BPH) preferred to feed on *hpl3-1* plants over WT plants, and female adults laid more eggs on *hpl3-1* plants ([Tong et al., 2012](#_ENREF_32)). However, mass of individual striped stem borer (SSB) larva was reduced in *hpl3-1* mutant plants. GLV-deficient *N. attenuata* plants slowed down the development of the chewing herbivore *Manduca sexta* larvae and decreased attractiveness to three lepidopteran herbivores, the closely related specialist species *Manduca sexta* and *M. quinquemaculata* and the generalist, *Spodoptera exigua* ([Halitschke et al., 2004](#_ENREF_11)). Field experiments with tobacco (*N. attenuata*) showed that GLV-producing wild type plants were more attractive to flea beetles (*Epitrix hirtipennis*) compared to plants with reduced *HPL* expression (as-*hpl*) ([Halitschke et al., 2004](#_ENREF_11)). The diverse effects of GLVs on different herbivores highlight the complexity of plant-herbivore interactions and the importance of understanding the specific context in which GLVs function in plant defense.

The whiteﬂy *Bemisia tabaci* (Hemiptera: Aleyrodidae), a typical phloem-feeding insect, is a species complex including invasive cryptic species such as the Middle East-Asia Minor (MEAM1) (De Barro *et al.,* 2011), which causing great economic losses around the world not only by direct sucking of plant sap but also by indirect transmission of devasting plant virus, such as African Cassava Mosaic virus ([Simmons and Riley, 2021](#_ENREF_30)). Here, we found that tobacco GLVs are vital for host adaption of whiteflies. Through integration of over-expression and virus-induced gene silencing (VIGS) approaches, we observed that higher accumulation of GLVs in tobacco plants with overexpressed *HPL* gene (*Hydroperoxide lyase*) was favorable for whitefly infestation, as indicated by higher survival rates and increased eggs laying on *HPL-OE* plants. Lower accumulation of GLVs in plants with *hpl*- and *adh*-silencing resulted in the reduced whitefly performance. Additionally, our findings demonstrated that emission of GLVs can affect the whiteflies^,^ preference to host. Taken together, we provide solid evidence for the significant contribution of GLVs in shaping plant-insect interactions.

**Materials and Methods**

**Plants**

The tabacco *Nicotiana tabacum* cv. NC89 was used in this study. To reduce the gene expression, 500 bp sequence from *HPL* or *ADH* were amplified and inserted into 2mDNA1 vector. The plasmids were transformed into *Agrobacterium tumefaciens* strain EHA105. The detailed construction method was shown below. Tobacco plants with 2-3 leaves were inoculated with HPL-2mDNA1 or ADH-2mDNA1 and cultivated in greenhouse (light: 06:00–20:00, 14:10 L/D, controlled temperature 25±3 °C, and relative humidity 60-80%) for 3 weeks. Then, the gene transcript abundance was determined and plants with higher silencing efficiency were selected to perform bioassay. The plants with empty vector 2mDNA1 were used as control. For construction of *HPL* gene overexpressed plants, pCHF3 recombinant plasmid containing full length HPL gene were transformed into plants. The callus with kanamycin resistance were cultivated into seedlings and verified by PCR and qRT-PCR.

**Whitefly culture**

The whitefly species, *Bemisia tabaci* MEAM1 (*mtCOI* GenBank accession no. GQ332577), from Zhejiang, China, was collected from eggplants in Rui’an (27°48′20′′N, 120°39′57′′E) in September 2008. In the experiments, whiteflies were maintained on healthy tobacco plants in insect proof cages (40cm× 50cm× 50cm) in climate-controlled rooms at 25±1 °C, 14L:10D light cycle, and 60–80 % RH.

**Subcellular localization analysis**

The full length of HPL was inserted into a modified pCambia1305-RFP vector and transformed into *Agrobacterium tumefaciems* strain GV3101*.* The images were taken 3 days after inoculation. The CLSM data were collected at the Bioimaging Center, State Key Laboratory for Managing Biotic and Chemical Threats to the Quality and Safety of Agro-products, Institute of Plant Virology, Ningbo University, Ningbo, China.

**Analysis of HPL activity**

HPL activity was analyzed with plant HPL ELISA Kit. HPL activity was determined using a standard curve equation (Y = 0.02534*X - 0.01446, R^2^ = 0.9984), followed by normalization with respect to a control sample.

**Analyses of HPL-derived metabolites**

Volatiles and endogenous HPL-derived metabolites were extracted and analyzed by GC–MS as described previously ([Chehab et al., 2008](#_ENREF_5)). Leaf samples were collected and frozen in liquid nitrogen. The materials were ground and a weighed amount of the sample was introduced into a 4 mL screw-top Supelco vial containing 500 μL of 1% NaCl. The vial was then rapidly capped with the screw top having a polytetrafluoroethylene/silicone septum, and incubated for 30 min in a water bath at 50°C. A 60 μm polydimethylsiloxane (PDMS)-coated solid phase micro extraction (SPME; Supelco) was used to measure the aldehydes released from the plant tissue. Measurements were done in triplicates. The headspace was sampled for 30 min with the PDMS-SPME and analyzed by GC-MS. GC-MS analysis was performed using a Hewlett and Packard 6890 series gas chromatograph coupled to an Agilent Technologies 5973 network mass selective detector. An HP-5MS column (30 m × 0.25 mm, 0.25 μm film thickness) was used with He (37 kPa) as carrier gas. The GC oven temperature was programmed as follows: 5 min 40°C, ramp to 225°C at 15°C/min and no hold time. Mass spectra in the electron impact mode were generated at 70 eV. Injection was performed by thermal desorption of the SPME in the injector at 200°C using the splitless injection mode. The compounds were identified by comparing the GC retention times and mass spectra with those of authentic reference compounds. The headspace was analyzed as described above and peak areas (mass-to-charge ratios 82 and 98) were determined. The aldehydes were quantified subsequent to careful preparation of calibration curves with different standards.

**Host plant suitability assays**

Five female and five male whitefly adults emerging within three days were collected from uninfected tobacco plants and released into a clip cage that was secured to the abaxial surface of a plant leaf (third to fifth leaf from the top). Each plant was equipped with three clip cages and the experiment was replicated thirty times. The number of adult whiteflies and eggs laid by whiteflies on each plant were counted after 7 days. Based on these data, the survival rate of adult whiteflies as well as the number of eggs laid per female per day were determined to assess host plant suitability.

**GLVs treatments**

Plants were grown for 22 days under the conditions mentioned above before being treated with GLVs. For the GLVs treatment, 10 plants in single pots were placed into airtight glass insect cage and were treated with 500 nmol of (z)-3-hexenol in lanolin. Control plants were smeared with the same volume of pure lanolin paste. The host plant suitability assays were conducted as above.

**Choice experiments**

The choice experiments were performed as described previously ([Li et al., 2017](#_ENREF_19)). Two plants with similar size and same leaf numbers were pretreated with or without [GLVs](https://www.ncbi.nlm.nih.gov/pmc/articles/PMC4311212/#def6) and placed in a cage (30 × 30 × 30 cm). Two hundred adult whiteflies were captured, placed on ice for 1 min, and then released onto the petri dish in the middle of the two plants. 15 min after insect release, the settled whiteflies were recaptured, and the number on each of the two plants was recorded.

**RNA extraction and RT-PCR analysis**

Total RNA was extracted from leaves with TRIzol (Invitrogen, USA). cDNA was synthesized from 1μg of total RNA using the SYBR^®^ PrimeScript RT-PCR Kit II (Takara Biotechnology, Dalian, China). qRT-PCR was performed using Bio-Rad CFX96™ Real-Time System (Bio-Rad, CA, USA). Eight independent biological samples and three technical repetitions were used. The average threshold cycle (Ct) was calculated per sample. The relative expression levels were calculated with the 2^-ΔΔCT^ method. The *glyceraldehyde-3-phosphate dehydrogenase* (*GAPDH*) gene was served as an internal control ([Zhang et al., 2012](#_ENREF_40)). Gene-specific primers used for examining the transcript abundance were listed in Supplementary Table 1.

**Virus-induced gene silencing (VIGS) and gene overexpressing plants assay**

Gene-specific primers for VIGS of *HPL* (Genebank accession no. DQ129870) and *ADH* (Genebank accession no. XM_016637202) genes were designed, which contain *BamH*I or *Xba*I enzyme digestion sites in the forward and reverse primer, respectively (Table 1). Fragments of *HPL* and *ADH* were amplified from tobacco cDNA. The PCR product was cloned into *Xba*I-*BamH*I-digested pBIN2mDNA1 plasmid, yielding a gene-silencing vector as previously described ([Huang et al., 2009](#_ENREF_16)). After sequencing confirmation of the fidelity for the insertion, the gene-silencing vector was transformed into *A. tumefaciens* strain EHA105 by electroporation. With the medical syringe, approximately, 0.2 mL of *A. tumefaciens* cultures (OD600 =0.8-1.0) that carry TbCSV as a helper virus and 2mDNA1-*HPL*/*ADH* constructs were co-infiltrated into the stem of each plant at the three-to-four true-leaf stage (VIGS silenced plants). Empty-vector plants were used as the control which were inoculated with *A. tumefaciens* cultures carrying TbCSV and pBIN2mDNA1. For gene overexpression, the *HPL* gene coding region was inserted into pCHF3 vector and transformed into *A. tumefaciens* strain EHA105 by electroporation. After that, leaf disk transformation of tobacco was performed and the callus was selected by Kanamycin. The F_0_ plants were used to determine the gene expression levels and host plant suitability assays. As described above, all plants were cultivated in a greenhouse under the same conditions. Total RNA was isolated from the third leaf from the top and then the silencing efficiency was evaluated by qRT-PCR.

**Data analysis**

All percentage data (adult survival) were arcsine square root transformed before statistical analysis. Statistical significance was evaluated using one-way ANOVA Student's *t*-test at a 0.05 level followed by least significant difference tests for comparisons of survival, the number of eggs laid and gene expression. All data analyses were conducted using SPSS 20.0 Statistics (IBM, Armonk, NY, USA).

**Results**

**HPL contributes plant adaption to whiteflies**

Hydroperoxide lyase (HPL), one of the important enzymes for GLVs synthesis, cleaves the C-C bond adjacent to the hydroperoxy group in the products of LOX pathway, resulting in the formation of C6 or C9 aldehydes that can undergo isomerization or dehydrogenation ([Mosblech et al., 2009](#_ENREF_24)). The RNA-seq data obtained from our laboratory (Luan et al., 2013) showed that the transcript level of *HPL* gene was significantly increased following whitefly infestation (Data were not shown). This observation suggests that the *HPL* might be responsive to whitefly resistance. Evolutionary analysis of *HPL* gene in tobacco indicated that it was present as a single copy in the genome (Figure 1A). To gain a deeper understanding of expression patterns of *HPL*, we conducted quantitative real-time PCR (qRT-PCR) experiment, and revealed that its expression was notably higher in leaves compared to those in roots and stems (Figure 1B). Furthermore, our investigation into the subcellular localization of the HPL protein demonstrated that it was primarily expressed in the chloroplasts of the plant cells (Figure 1C).

To investigate the potential role of HPL in plant defense against whiteflies, we conducted a series of experiments. First, we compared the relative transcript levels of *HPL* gene in plants with or without whitefly infestation. Remarkably, we observed a significant upregulation of *HPL* gene expression at the early stage of whitefly infestation (Figure 2A), suggesting that HPL might play a crucial role in plant resistance to whiteflies. We also observed slightly increase of HPL activity after whitefly infestation (Figure 2B). Then, we generated transgenic tobacco plants with overexpressed *HPL* gene (*HPL-OE*). No obvious growth phenotypic differences were observed between *HPL-OE* plants and wild-type plants. The transcript level of *HPL* in these transgenic plants was remarkably higher, approximately 60 times more than that in wild-type plants, which led to a remarkable increase for enzyme activity (Figure 2C-D). The survival rate of whiteflies fed on *HPL-OE* plants was significantly higher compared to those feeding on control plants (Figure 2E). Furthermore, female adults presented increased fecundity, laying approximately 1.53-fold more eggs per day compared to those feeding on wild-type plants (Figure. 2F). To further validate these findings, bioassay on *HPL* gene silencing (*hpl-VIGS*) and control plants was performed. Seven days after releasing adult whiteflies to the plants, the survival rate of whiteflies on *hpl-VIGS* plants was slightly lower than that on empty-vector-inoculated plants, although the difference was not statistically significant (Fig. 2G-I). However, the mean number of eggs laid per day by per female on *hpl-VIGS* plants was significantly lower (Figure 2J).

Taken together, these results suggest that HPL plays a critical role in determining the suitability of host plants to whiteflies.

**HPL mediated GLVs biosynthesis is associated with plant defense**

As *HPL* gene encodes a GLV biosynthetic enzyme, manipulating its expression through overexpression or silencing led to alterations of its catalytic activity. To validate the impact of HPL overexpression or silencing on GLVs release, we quantified the amounts of the GLVs with high accumulation after insect attack, including hexanal, 1-hexanal, trans-2-hexanal, and cis-3-hexanal, in the plants. The results revealed that in the HPL-overexpressing (*HPL-OE*) plants, the content of all the indicated GLVs compounds was markedly increased, reaching approximately two times that of the control plants (Figure 3A). In contrast, silencing of the *HPL* gene led to a significant reduction in the release of GLVs (Figure 3B). All the data support the conclusion that HPL plays a critical role in manipulating the GLV aldehyde pools in the plant.

To directly investigate the role of GLVs on whitefly performance, we conducted an exogenous application experiment with three representative GLVs. Both 500 nmol/L trans-2-hexenal and cis-3-hexenol treatment led to reduced plant defense against whiteflies. Whiteflies exhibited higher survival rates and laid more eggs on plants treated with these GLVs (Figures 4A&B). Although application of cis-3-hexenyl acetatet slightly promoted whitefly performance, the difference has no statistical difference (Figure 4C). Taken together, our results demonstrated that GLVs negatively regulate plant defense against whiteflies.

**GLVs establish the host selection for whitefly**

Previous studies have suggested that GLVs could impact the preference of insects to specific plants. Thus, we speculated that changes in GLVs release might also alter flying orientations of whiteflies. To test this hypothesis, we examined the choice behavior of female and male whiteflies to wild type and *HPL-OE* plants and found that whiteflies preferred to the plants with high GLV accumulation (Figure 5A). In contrast, *HPL* gene silencing plant became noticeably less attractive to female and male whiteflies (Figure 5B). Then, we conducted a comparison of whitefly preferences to cis-3-hexenol treated or untreated plants. Remarkably, the cis-3-hexenol treated plants exhibited a significantly higher preference for whiteflies. The percentage of whiteflies on cis-3-hexenol treated plants was approximately four times higher than that on the control plants (Figure 5C). Similarly, trans-2-hexenal treatment also had a notable impact on whitefly preference. The choice probability for whiteflies to trans-2-hexenal treated plant was remarkably high, ranging from 75% to 90% (Figure 5D). Taken together, the results indicate that plants GLVs is associated with defense against whiteflies.

**Alcohol dehydrogenase contributes whitefly adaption to tobacco**

Metabolites produced by HPL is quite unstable and can be isomerized to the corresponding higher stable alcohol by alcohol dehydrogenase (ADH) ([Scala et al., 2013a](#_ENREF_27)). To further confirm the role of HPL pathway in plant suitability to whitefly, we investigated the performance of whiteflies on *adh-VIGS* tobacco. Through qRT-PCR analysis, we observed a significant reduction in the transcript levels of *ADH* in *adh-VIGS* plants, reaching only 38% of the levels found in the empty-vector plants (Figure 6A). Seven days after whitefly infestation, the survival rate of whiteflies on *adh-VIGS* plants was lower than that on control plants (Figure 6B). Adult female whiteflies laid a reduced number of eggs on *adh-VIGS* tobacco (Figure 6C).

**Discussion**

As plants are sessile organisms and continuously subjected to a wide array of biotic and abiotic challenges, they arm themselves with numerous constitutive and inducible defense mechanisms against attackers ([Chen and Mao, 2020](#_ENREF_6); [Hancock et al., 2015](#_ENREF_14)). Currently, the phytohormone has garnered extensive attention, especially JA, salicylic acid and ethylene ([Erb and Reymond, 2019](#_ENREF_8); [Ferry et al., 2004](#_ENREF_10); [Meiners, 2015](#_ENREF_23); [Zebelo and Maffei, 2015](#_ENREF_38)). Green leaf volatiles (GLVs) and jasmonates (JAs) are the most widely distributed oxylipin compound found throughout the plant kingdom. JA has undergone extensive research due to its pivotal role in plant resistance against insect herbivores. Conversely, the role of GLVs in plant defense remains a subject of debate, as they appear to serve varying functions in response to different herbivores. In the present study, we clarified the significant role of the GLV/HPL pathway in plant defense.

GLVs are emitted in trace amount when plant tissues are intact, but are rapidly released within seconds or minutes upon mechanical wounding, herbivore attack, or abiotic stress ([Halitschke et al., 2004](#_ENREF_11); [Turlings et al., 1995](#_ENREF_33)). Given that GLV/HPL products could be induced by whiteflies and other herbivores (Tong et al.,2012; Wang et al., 2015), it seems that plants posses the ability to perceive insect infestation and subsequently activate the HPL pathway as part of their defense response. Here, we demonstrated that HPL pathway plays a negative role in tobacco response to sucking insects whiteflies. Whiteflies performed better on *HPL-OE* tobacco plants compared to wild type (Figures 2D-F). Conversely, silencing of *NtHPL* decreased plant adaptability to whiteflies, leading to reduced survival rate and fecundity of whiteflies (Figures 2H-J). However, previous reports have shown that function of HPL in plant direct defense against herbivore can vary widely, possibly depending on the insect or host plant species. For example, *Manduca quinquemaculata*, *Spodoptera exigua* larvae and *Manduca sexta* neonates preferred to choose excised leaves of wild type *Nicotiana attenuata* (WT), consumed wider leaf area and grew significantly faster than that on *as-hpl*plants ([Halitschke et al., 2004](#_ENREF_11)). In contrast, OsHPL3 positively modulates resistance to rice brown planthopper but negatively modulates resistance to the rice striped stem borer and white-backed planthopper ([Liu et al., 2012](#_ENREF_21); [Tong et al., 2012](#_ENREF_32)). Depletion of *HPL* in potatoes promoted aphid performance ([Vancanneyt et al., 2001](#_ENREF_34)). Nevertheless, study on phloem-feeding insect (aphids: *Myzus persicae*) and an insect herbivore (leafminers: *Liriomyza trifolii*) showed no significant differences in performance between WT and *HPL-OE* arabidopsis plants ([Chehab et al., 2008](#_ENREF_5)).

As HPL is a key enzyme controlling the synthesis of GLVs, the alternation of its transcript might change the release of GLVs, such as hexanal, 1- hexanal, trans-2- hexanal and cis-3- hexanal. Here, we showed that overexpression of *HPL* promoted the release of GLVs, while silencing of *HPL* reduced their accumulation (Figure. 3). Exogenous application of (*Z*)-3-hexenol also indicated GLVs play a role in facilitating whitefly performance (Figure 4). These findings indicate that the individual metabolic component in GLVs could mediate plant resistance to herbivores. Previous studies have also explored the effects of GLVs on insect performance. For example, treatment of tea plants with (*Z*)-3-hexenol reduced the performance of tea geometrid *Ectropis obliqua* by interfering with JA and ethylene (ET) pathway (Xin et al., 2016). Exposure to (Z)-3-hexenol vapors in the glass jars decreased weight gain and oviposition of *B. tabaci* and shorten the total feeding period and phloem ingestion and increased the frequency of stylet puncture in tomato (Yang et al, 2020).

Plant HPL pathway plays a crucial role in regulating foraging behavior of insects. Y-tube and greenhouse experiments showed plastic dummies baited with either single compounds or GLV mixtures were more attractive to tea aphid compared to hexane baited controls ([Han and Chen, 2002](#_ENREF_13)). The adult emerald ash borer, *Agrilus planipennis*, was more attractive to the (*Z*)-3-hexenol containing purple prism traps (Grant et al., 2010). In addition, the mixture of C6-volatiles ((*Z*)-3-hexenol and (*Z*)-3-hexenyl acetate) and benzaldehyde in a natural ratio was found more attractive to female fruit moth *Cydia molesta* (Natale et al., 2003). Whiteflies, an insect with flying capabilities, could chose the host plants based on their preferences. In this study, we found that tobacco plants with GLVs application become more attractive to whiteflies, further confirming that HPL pathway plays a vital role in whitefly and plants interaction.

GLV production may itself be used as a signal by plants to coordinate their defensive response to herbivores. Here, we shed light on the complex interactions between plants and insects, particularly the role of GLVs in influencing insect behavior and host plant selection .These insights provide valuable knowledge that may aid in the development of novel strategies for enhancing plant resistance to herbivores.

**Consent for publication：**The work described has not been published before and its publication has been approved by all co-authors.

**Availability of data and material:** The data that support the findings of this study are openly available.

**Acknowledgements:** This work was supported by National Key R&D Program of China (2022YFD1401200, 2021YFC2600104) and the earmarked fund for China Agriculture Research System (CARS-23-C05).

**Conflict of interests**: The authors declare that they have no conflict of interests.

**Ethics declarations：**The authors declare that the research project has been conducted ethically, keeping in mind privacy, consent and appropriate reporting.

**Author Contributions:**

W.H.D carried out most of the experiments. P.L. designed and helped to carry out the experiments, analyzed data and wrote the first draft of the manuscript. C.L helped extract the plant RNA and performed bioassays of whiteflies. S.S.H helped to measure the content of GLVs. Y.Z.T helped to take confocal images. Y.Q.L conceived and supervised the study and revised the manuscript.

**Reference**

Allmann, S., and Baldwin, I.T. (2010). Insects betray themselves in nature to predators by rapid isomerization of green leaf volatiles. Science *329*, 1075-1078.

Ameye, M., Allmann, S., Verwaeren, J., Smagghe, G., Haesaert, G., Schuurink, R.C., and Audenaert, K. (2018). Green leaf volatile production by plants: a meta-analysis. New Phytol *220*, 666-683.

Ameye, M., Audenaert, K., De Zutter, N., Steppe, K., Van Meulebroek, L., Vanhaecke, L., De Vleesschauwer, D., Haesaert, G., and Smagghe, G. (2015). Priming of wheat with the green leaf volatile Z-3-hexenyl acetate enhances defense against *Fusarium graminearum* but boosts deoxynivalenol production. Plant Physiol *167*, 1671-1684.

Brodmann, J., Twele, R., Francke, W., Holzler, G., Zhang, Q.H., and Ayasse, M. (2008). Orchids mimic green-leaf volatiles to attract prey-hunting wasps for pollination. Curr Biol *18*, 740-744.

Chehab, E.W., Kaspi, R., Savchenko, T., Rowe, H., Negre-Zakharov, F., Kliebenstein, D., and Dehesh, K. (2008). Distinct roles of jasmonates and aldehydes in plant-defense responses. PLoS One *3*, e1904.

Chen, C.Y., and Mao, Y.B. (2020). Research advances in plant-insect molecular interaction. F1000Research *9*.

Clavijo McCormick, A., Unsicker, S.B., and Gershenzon, J. (2012). The specificity of herbivore-induced plant volatiles in attracting herbivore enemies. Trends Plant Sci *17*, 303-310.

Erb, M., and Reymond, P. (2019). Molecular interactions between plants and insect herbivores. Annu Rev Plant Biol *70*, 527-557.

Farag, M.A., and Pare, P.W. (2002). C6-Green leaf volatiles trigger local and systemic VOC emissions in tomato. Phytochemistry *61*, 545-554.

Ferry, N., Edwards, M.G., Gatehouse, J.A., and Gatehouse, A.M. (2004). Plant-insect interactions: molecular approaches to insect resistance. Curr Opin Biotechnol *15*, 155-161.

Grant, G. G., Ryall, K. L., Lyons, D. B., & Abou‐Zaid, M. M. (2010). Differential response of male and female emerald ash borers (Col., Buprestidae) to (Z)‐3‐hexenol and manuka oil. J. Appl. Entomol*.* *134*(1), 26-33.

Halitschke, R., Ziegler, J., Keinanen, M., and Baldwin, I.T. (2004). Silencing of hydroperoxide lyase and allene oxide synthase reveals substrate and defense signaling crosstalk in *Nicotiana attenuata*. Plant J *40*, 35-46.

Hammerbacher, A., Coutinho, T.A., and Gershenzon, J. (2019). Roles of plant volatiles in defence against microbial pathogens and microbial exploitation of volatiles. Plant Cell Environ *42*, 2827-2843.

Han, B.Y., and Chen, Z.M. (2002). Composition of the volatiles from intact and mechanically pierced tea aphid-tea shoot complexes and their attraction to natural enemies of the tea aphid. J Agric Food Chem *50*, 2571-2575.

Hancock, R.D., Hogenhout, S., and Foyer, C.H. (2015). Mechanisms of plant-insect interaction. J Exp Bot *66*, 421-424.

Hirao, T., Okazawa, A., Harada, K., Kobayashi, A., Muranaka, T., and Hirata, K. (2012). Green leaf volatiles enhance methyl jasmonate response in Arabidopsis. J Biosci Bioeng *114*, 540-545.

Huang, C., Xie, Y., and Zhou, X. (2009). Efficient virus-induced gene silencing in plants using a modified geminivirus DNA1 component. Plant Biotechnol J *7*, 254-265.

Jones, A.C., Cofer, T.M., Engelberth, J., and Tumlinson, J.H. (2022). Herbivorous caterpillars and the green leaf volatile (GLV) quandary. J Chem Ecol *48*, 337-345.

Joo, Y., Schuman, M.C., Goldberg, J.K., Wissgott, A., Kim, S.G., and Baldwin, I.T. (2019). Herbivory elicits changes in green leaf volatile production via jasmonate signaling and the circadian clock. Plant Cell Environ *42*, 972-982.

Li, P., Shu, Y.N., Fu, S., Liu, Y.Q., Zhou, X.P., Liu, S.S., and Wang, X.W. (2017). Vector and nonvector insect feeding reduces subsequent plant susceptibility to virus transmission. New Phytol *215*, 699-710.

Liu, J., Sun, L., Fu, D., Zhu, J., Liu, M., Xiao, F., and Xiao, R. (2022). Herbivore-induced rice volatiles attract and affect the predation ability of the wolf spiders, *Pirata subpiraticus* and *Pardosa pseudoannulata*. Insects *13*.

Liu, X., Li, F., Tang, J., Wang, W., Zhang, F., Wang, G., Chu, J., Yan, C., Wang, T., Chu, C.*, et al.* (2012). Activation of the jasmonic acid pathway by depletion of the hydroperoxide lyase OsHPL3 reveals crosstalk between the HPL and AOS branches of the oxylipin pathway in rice. PLoS One *7*, e50089.

Loughrin, J.H., Manukian, A., Heath, R.R., Turlings, T.C., and Tumlinson, J.H. (1994). Diurnal cycle of emission of induced volatile terpenoids by herbivore-injured cotton plant. Proc Natl Acad Sci U S A *91*, 11836-11840.

Luan, J. B., Yao, D. M., Zhang, T., Walling, L. L., Yang, M., Wang, Y. J., Liu, S. S. (2013). Suppression of terpenoid synthesis in plants by a virus promotes its mutualism with vectors. Ecology Letters, 16(3), 390-398.

Meiners, T. (2015). Chemical ecology and evolution of plant-insect interactions: a multitrophic perspective. Curr Opin Insect Sci *8*, 22-28.

Mosblech, A., Feussner, I., and Heilmann, I. (2009). Oxylipins: structurally diverse metabolites from fatty acid oxidation. Plant Physiol Biochem *47*, 511-517.

Najdabbasi, N., Mirmajlessi, S.M., Dewitte, K., Ameye, M., Mand, M., Audenaert, K., Landschoot, S., and Haesaert, G. (2021). Green leaf volatile confers management of late blight disease: a green vaccination in potato. Journal of fungi *7*.

Natale, D., Mattiacci, L., Hern, A., Pasqualini, E., & Dorn, S. (2003). Response of female *Cydia molesta* (Lepidoptera: Tortricidae) to plant derived volatiles. Bull. Entomol. Res., *93*(4), 335-342.

Paudel Timilsena, B., Seidl-Adams, I., and Tumlinson, J.H. (2020). Herbivore-specific plant volatiles prime neighboring plants for nonspecific defense responses. Plant Cell Environ *43*, 787-800.

Scala, A., Allmann, S., Mirabella, R., Haring, M.A., and Schuurink, R.C. (2013a). Green leaf volatiles: a plant's multifunctional weapon against herbivores and pathogens. Int J Mol Sci *14*, 17781-17811.

Scala, A., Mirabella, R., Mugo, C., Matsui, K., Haring, M.A., and Schuurink, R.C. (2013b). E-2-hexenal promotes susceptibility to *Pseudomonas syringae* by activating jasmonic acid pathways in Arabidopsis. Front Plant Sci *4*, 74.

Shiojiri, K., Kishimoto, K., Ozawa, R., Kugimiya, S., Urashimo, S., Arimura, G., Horiuchi, J., Nishioka, T., Matsui, K., and Takabayashi, J. (2006). Changing green leaf volatile biosynthesis in plants: an approach for improving plant resistance against both herbivores and pathogens. Proc Natl Acad Sci U S A *103*, 16672-16676.

Simmons, A.M., and Riley, D.G. (2021). Improving whitefly management. Insects *12*.

Takabayashi, J., and Shiojiri, K. (2019). Multifunctionality of herbivory-induced plant volatiles in chemical communication in tritrophic interactions. Curr Opin Insect Sci *32*, 110-117.

Tong, X., Qi, J., Zhu, X., Mao, B., Zeng, L., Wang, B., Li, Q., Zhou, G., Xu, X., Lou, Y.*, et al.* (2012). The rice hydroperoxide lyase OsHPL3 functions in defense responses by modulating the oxylipin pathway. Plant J *71*, 763-775.

Turlings, T.C., Loughrin, J.H., McCall, P.J., Rose, U.S., Lewis, W.J., and Tumlinson, J.H. (1995). How caterpillar-damaged plants protect themselves by attracting parasitic wasps. Proc Natl Acad Sci U S A *92*, 4169-4174.

Vancanneyt, G., Sanz, C., Farmaki, T., Paneque, M., Ortego, F., Castanera, P., and Sanchez-Serrano, J.J. (2001). Hydroperoxide lyase depletion in transgenic potato plants leads to an increase in aphid performance. Proc Natl Acad Sci U S A *98*, 8139-8144.

Wasternack, C., and Feussner, I. (2018). The oxylipin pathways: Biochemistry and function. Annu Rev Plant Biol *69*, 363-386.

Xin, Z., Ge, L., Chen, S., and Sun, X. (2019). Enhanced transcriptome responses in herbivore-infested tea plants by the green leaf volatile (Z)-3-hexenol. J Plant Res *132*, 285-293.

Xin, Z., Li, X., Li, J., Chen, Z., & Sun, X. (2016). Application of chemical elicitor (Z)-3-hexenol enhances direct and indirect plant defenses against tea geometrid *Ectropis obliqua*. *BioControl*, *61*(1), 1-12.

Yang, F., Zhang, Q., Yao, Q., Chen, G., Tong, H., Zhang, J., ... & Zhang, Y. (2020). Direct and indirect plant defenses induced by (Z)-3-hexenol in tomato against whitefly attack. J PEST SCI, *93*(4), 1243-1254.

Ye, M., Glauser, G., Lou, Y., Erb, M., and Hu, L. (2019). Molecular dissection of early defense signaling underlying volatile-mediated defense regulation and herbivore resistance in rice. Plant Cell *31*, 687-698.

Zebelo, S.A., and Maffei, M.E. (2015). Role of early signalling events in plant-insect interactions. J Exp Bot *66*, 435-448.

Zhang, P.J., Wei, J.N., Zhao, C., Zhang, Y.F., Li, C.Y., Liu, S.S., Dicke, M., Yu, X.P., and Turlings, T.C.J. (2019). Airborne host-plant manipulation by whiteflies via an inducible blend of plant volatiles. Proc Natl Acad Sci U S A *116*, 7387-7396.

Zhang, T., Luan, J.B., Qi, J.F., Huang, C.J., Li, M., Zhou, X.P., and Liu, S.S. (2012). Begomovirus-whitefly mutualism is achieved through repression of plant defences by a virus pathogenicity factor. Molecular Ecology *21*, 1294-1304.

Zogli, P., Pingault, L., Grover, S., and Louis, J. (2020). Ento(o)mics: the intersection of 'omic' approaches to decipher plant defense against sap-sucking insect pests. Curr Opin Plant Biol *56*, 153-161.

**Figure legends**

**
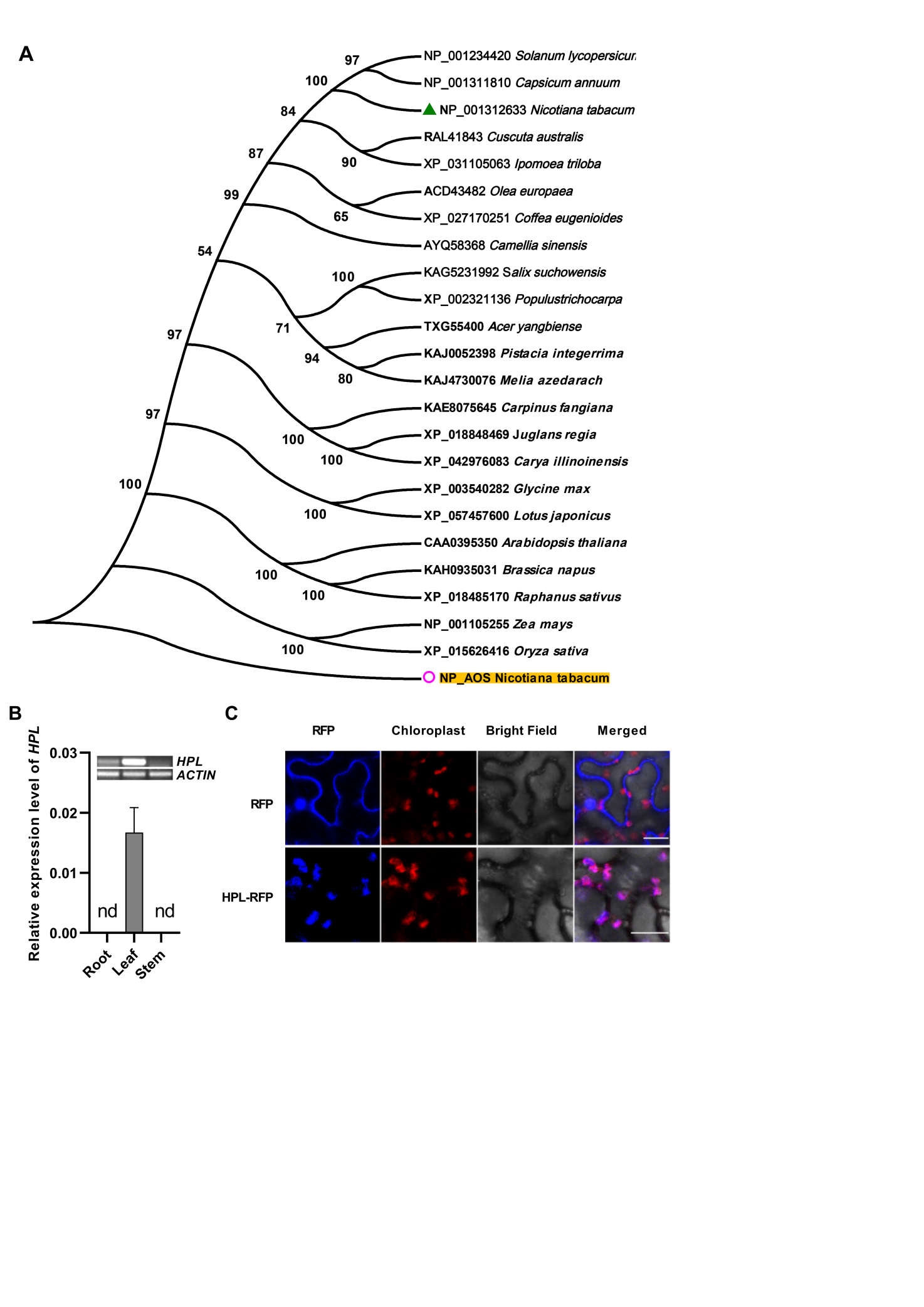
**

**Figure 1 Characterization of HPL in tobacco.**

1. Phylogenetic analysis of HPL among different plant species. Neighbor-Joining method was recruited and a bootstrap analysis of 1000 replicates was used. The resulting bootstrap values were shown at the nodes in the cladogram. The phylogenetic analysis was performed with MEGA5 software. Serial numbers of the proteins used in the figure for different plant species were marked before the respective plant names. AOS was identified as the most homologous protein and served as the outgroup for the evolutionary tree. (B) Transcripts of *HPL* gene in root, stem and leaf determined by qRT-PCR and RT-PCR. nd indicated no Ct values were determined by qRT-PCR. PCR cycle for RT-PCR was 45. (C) Subcellular localization of HPL protein. HPL was fused with RFP (red fluorescent protein) to observed the subcellular localization, while the chloroplast was marked with its autofluorescence.


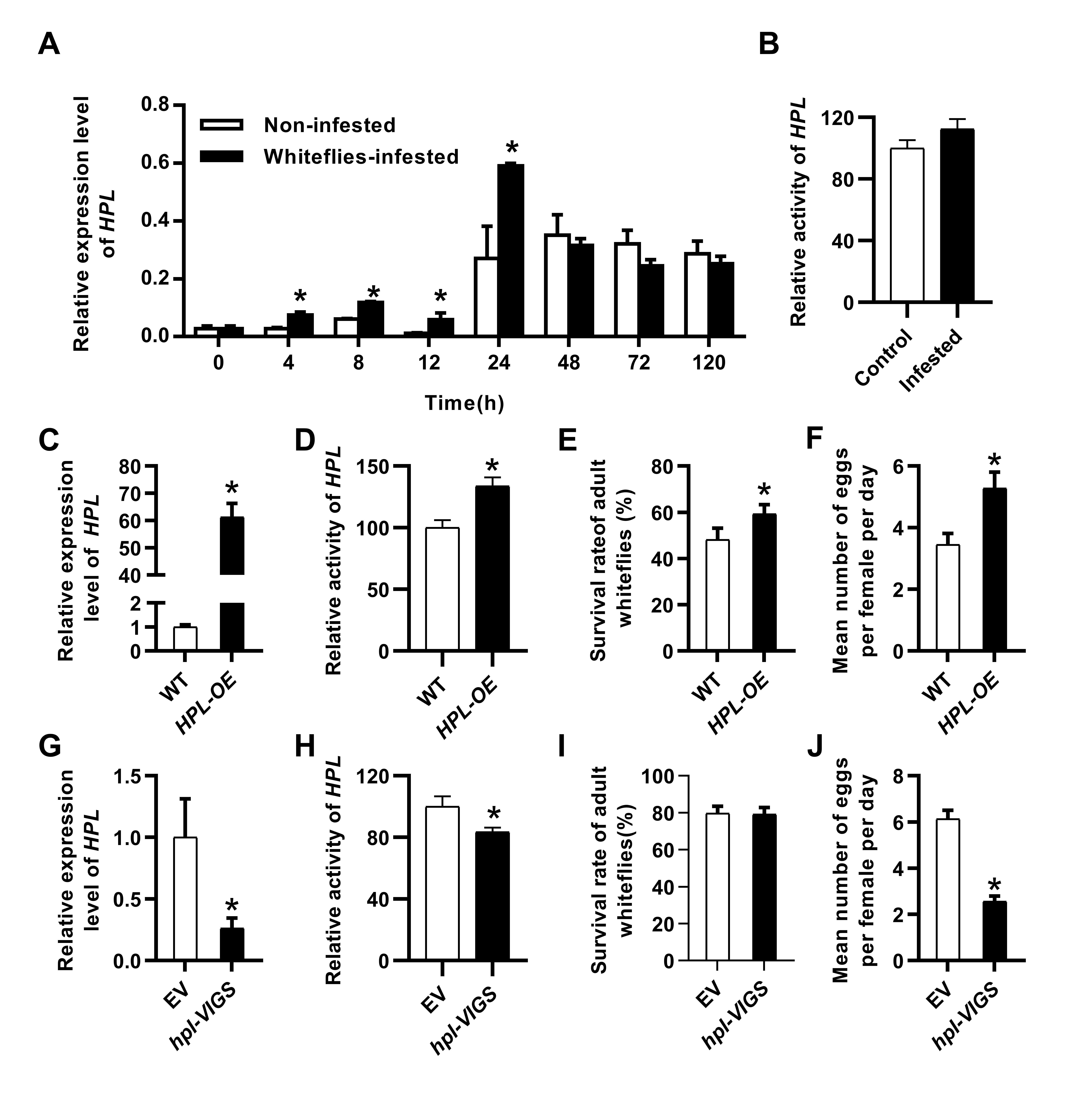


**Figure 2 HPL is associated with plant defense against whiteflies**

1. Relative transcript level of *HPL* to GADPH (glyceraldehyde-3-phosphate dehydrogenase) in whiteflies infested and control plants at the indicated time points. (B) Enzyme activity of HPL 24 h after whitefly infestation. HPLactivity for control plants was set as 100%. (C-D) Relative transcript level and activity of HPL in transgenic plants and control. Gene expression or enzyme activity in WT was set as 1 or 100%. (E-F) Performance of whiteflies on *HPL-OE* plants. The whiteflies are allowed to feed on control and transgenic tobacco plants for seven days. The survival rates of the adults (E) or the number of eggs/female/day (F) on transgenic and control plants were calculated. (G-H) Expression and enzyme activity of HPL in *hpl-VIGS* plants. Gene expression or enzyme activity in WT was set as 1 or 100%. (I-J) Performance of whiteflies on *hpl-VIGS* plants. Seven days after whiteflies feeding, survival rates of whiteflies (I) and mean number of eggs laid by per female adult per day (J) on *hpl-VIGS* and empty vector-inoculated control plants. Data are shown as mean ±SE, n = 3 ( B, D, H), 8 (A, C, G) or 30 (E-F, I-J). Asterisk above the bars indicates significant differences between treatments (*P* < 0.05, Student’s t-test).

**Figure 3 HPL mediates GLVs** **synthesis.**

Amount of Hexanal, 1-Hexanal, Trans-2- Hexanal and Cis-3- Hexanal (cis-3-hexen-1-ol) in control, *HPL* overexpressed (*HPL-OE*) or *hpl-VIGS* (B) plants were measured. Data shown are mean ± SE. Asterisk above the bars indicates significant differences between treatments (*P* < 0.05, Student’s t-test).

**Figure 4 Exogenous application of GLVs could facilitate whitefly performance.** Seven days after whitefly feeding, the survival rate of the adults and the number of eggs laid by per female per day on tobacco plants that were individually treated with 500 nmol of trans-2-hexenal (A), cis-3-hexenol (B) or cis-3-haxenyl acetate (C)were analyzed. Data shown are mean±SE, *n* = 30. Asterisk above the bars indicate significant differences between treatments (*P* < 0.05, Student’s t-test).


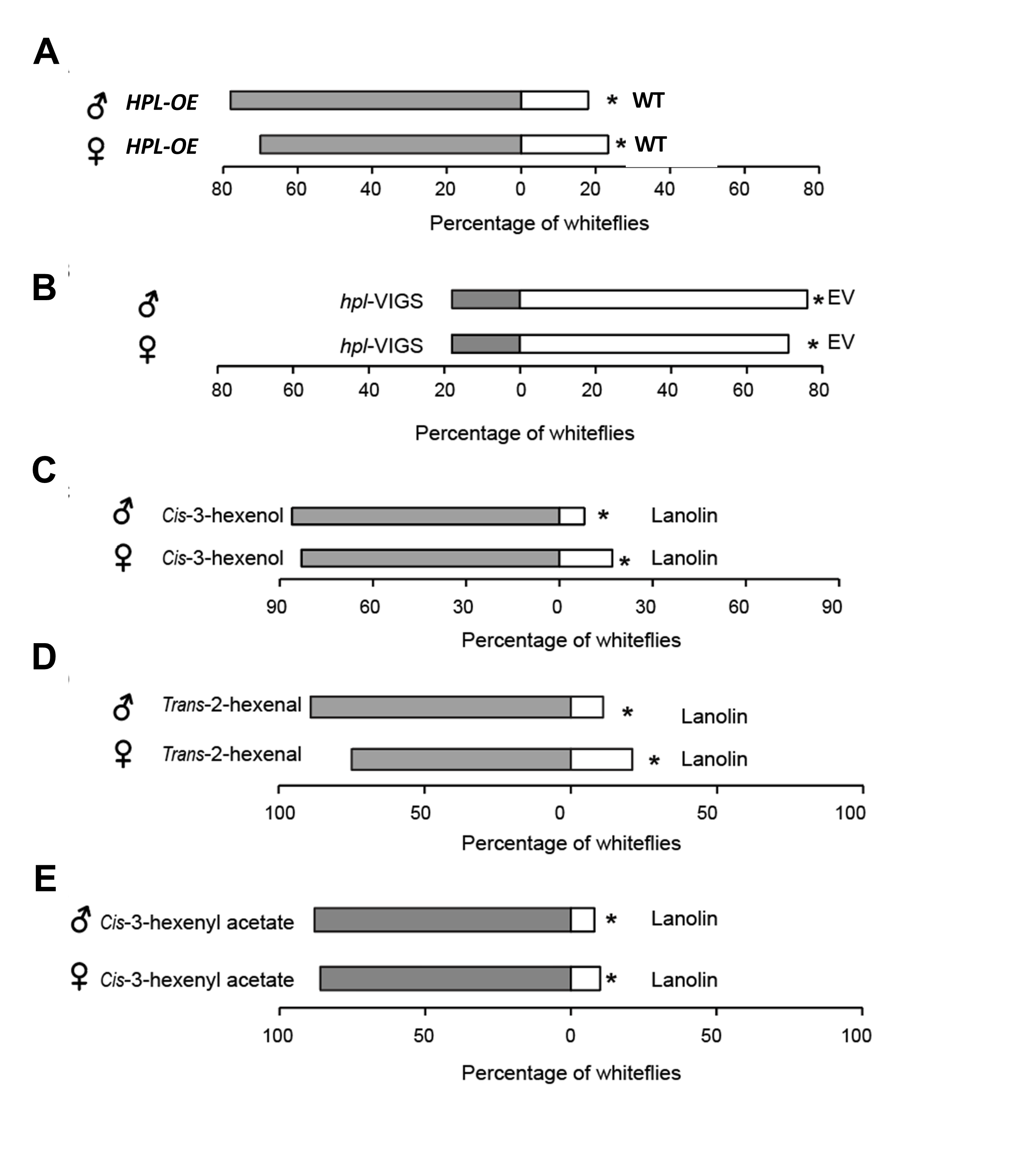


**Figure 5. GLVs play important role in host plant option.** (A&B), Host choice experiment for adult whiteflies to control, *HPL-OE* or *hpl-VIGS* plants. (C-E), Host plants choice experiment for adult whiteflies to control or plants with 500 nmol *cis*-3-hexenol (C), *trans*-2-hexenal (D) and *Cis*-3-hexenyl acetate (E). The whiteflies with no option were also taken into account. All experiment was repeated twice with similar results. Asterisk indicated significant difference (*P*<0.05, Wilcoxon matched pairs test)

**Figure 6 The performance of whiteflies on *adh-VIGS* and empty vector inoculated plants.** (A) Transcripts of *ADH* gene in *adh-VIGS* plants. Values are means±SE (*n*=8). Seven days after whiteflies feeding, the survival rates of the adults (B) and the number of eggs by per female per day (C) on *adh-VIGS* tobacco plants and empty vector inoculated plants were compared. Data shown are mean±SE, *n* = 30. Asterisk above the bars indicate significant differences between treatments (*P* < 0.05, Student’s t-test).

Supplementary Table

Table 1 Primers used in this study

| Gene | GenBank accession | Primer sequences (5’→3’) | Application |
| --- | --- | --- | --- |
| *HPL* | DQ129870 | AATGGCGAAAATGATGAGC  GAATTGTACGGACGGGAAG | qRT-PCR |
| *ADH*  *GAPDH* | XM_016637202  Z72488 | GCTTCTAGGGTCATTGGCAT  CATCTCAGCAATGACCTGCT  GCAGTGAACGACCCATTTATCTC  AACCTTCTTGGCACCACCCT | qRT-PCR  qRT-PCR |
| *HPL*  *ADH* | DQ129870  XM_016637202 | TGCTCTAGAACAAATAGCACCCCAAT  CGCGGATCCTTGACAAATCTTGGCCTTTG  CGCGGATCCGTGCCATACTGATGTTTACT  TGCTCTAGACACAACTTAGAACGCAAA | *HPL* VIGS  *ADH* VIGS |
| *HPL* | DQ129870 | CGCGGATCCATGTCCACAATAATGGCG  TGCTCTAGATCAACTGGCTTTTTTCACAG | HPL-OE |
| *HPL* | DQ129870 | AGCTCTAGAATGTCCACAATAATGGCG  CATGGATCCACTGGCTTTTTTCACAGA | HPL-RFP |
